# Cyclopropanol Warhead in Malleicyprol Confers Virulence of Human‐ and Animal‐Pathogenic *Burkholderia* Species

**DOI:** 10.1002/anie.201907324

**Published:** 2019-08-27

**Authors:** Felix Trottmann, Jakob Franke, Ingrid Richter, Keishi Ishida, Michael Cyrulies, Hans‐Martin Dahse, Lars Regestein, Christian Hertweck

**Affiliations:** ^1^ Department of Biomolecular Chemistry Leibniz Institute for Natural Product Research and Infection Biology (HKI) Beutenbergstr. 11a 07745 Jena Germany; ^2^ Institute of Organic Chemistry, BMWZ Leibniz University Hannover 30167 Hannover Germany; ^3^ Department Bio Pilot Plant Leibniz Institute for Natural Product Research and Infection Biology (HKI) 07745 Jena Germany; ^4^ Department Infection Biology Leibniz Institute for Natural Product Research and Infection Biology (HKI) 07745 Jena Germany; ^5^ Natural Product Chemistry Faculty of Biological Sciences Friedrich Schiller University Jena 07743 Jena Germany

**Keywords:** natural products, mass spectrometry, polyketides, structure elucidation, virulence factors

## Abstract

*Burkholderia* species such as *B. mallei* and *B. pseudomallei* are bacterial pathogens causing fatal infections in humans and animals (glanders and melioidosis), yet knowledge on their virulence factors is limited. While pathogenic effects have been linked to a highly conserved gene locus (*bur*/*mal*) in the *B. mallei* group, the metabolite associated to the encoded polyketide synthase, burkholderic acid (*syn*. malleilactone), could not explain the observed phenotypes. By metabolic profiling and molecular network analyses of the model organism *B. thailandensis*, the primary products of the cryptic pathway were identified as unusual cyclopropanol‐substituted polyketides. First, sulfomalleicyprols were identified as inactive precursors of burkholderic acid. Furthermore, a highly reactive upstream metabolite, malleicyprol, was discovered and obtained in two stabilized forms. Cell‐based assays and a nematode infection model showed that the rare natural product confers cytotoxicity and virulence.

## Graphical abstract

**Virulence factor**: The true effector molecules of a disease‐determining biosynthetic gene cluster from diverse pathogenic bacteria were discovered. Polyketides equipped with a cyclopropanol warhead show high chemical reactivity and strong potency in a eukaryotic infection model *Caenorhabditis elegans*. The chemical relationships between all the complex polyketides were decrypted.

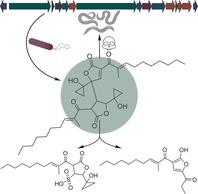

B*urkholderia mallei* and *Burkholderia pseudomallei* are closely related Gram‐negative bacteria that have become infamous for causing human and animal diseases with high lethality. Infections with *B. mallei*, transmitted from horses, lead to glanders, a highly contagious zoonotic disease which shows up to 95 % mortality when untreated, and even 50 % when treated with antibiotics.^1^ The soil‐ and water‐dwelling *B. pseudomallei* may infect various cell types and evade the immune system, causing melioidosis.^2^ This life‐threatening disease is an important cause of severe sepsis in Southeast Asia and Northern Australia, showing mortality rates of up to 40 %.^3^ Once infected, antibiotic treatment regimes against *B. pseudomallei* typically last longer than five months.^4^ Thus, melioidosis has been recognized as a growing threat to global health. Because of the low infective dose needed, the possibility of infection by inhalation, and the limited preparedness in most countries, *B. mallei* and *B. pseudomallei* have been classified as potential biological warfare agents.^5^ Previous studies have identified macromolecular virulence determinants^6^ such as the proteinogenic toxin *Burkholderia* lethal factor 1 (BLF1),^7^ and have implicated the involvement of secondary metabolites^8^ including the siderophores malleobactins^9^ in pathogenesis. Yet, in light of the high biosynthetic potential of these bacteria there is a clear gap in knowledge of small‐molecule virulence factors of these notorious pathogens. Particularly enigmatic is the function of a polyketide synthase encoded by the *bur/mal* gene locus (Figure 1 A), which is highly conserved in the genomes of all bacteria belonging to the *B. pseudomallei* group^10^ and in *B. contaminans*, an emerging pathogen in cystic fibrosis.^11^ Deletions in the *bur/mal* gene cluster reduced the virulence of *B. pseudomallei* 12 and its low‐pathogenicity model organism *B. thailandensis* 13 against the infection model *Caenorhabditis elegans*. However, the previously identified metabolite, malleilactone^13^ *syn*. burkholderic acid^14^ (**1**, Figure 1), could not explain the virulence associated to the presence of the corresponding biosynthetic gene cluster. Compound **1** exhibited only weak antiproliferative^14^ and moderate cytotoxic^13^ activities in eukaryotic cell line assays, and no effect on the fitness of *C. elegans* was observed.^13^ Hence, it was questioned whether **1** was the true virulence factor associated with the *bur* assembly line. Herein we report the discovery of previously overlooked cyclopropanol‐substituted polyketides originating from the *bur*‐encoded pathway and show that highly reactive precursors of **1** are the actual virulence factors, as demonstrated in the *C. elegans* infection model.

**Figure 1:**
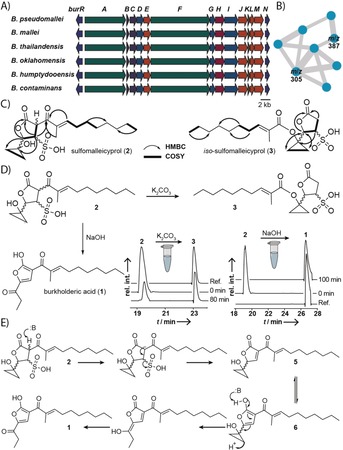
Conserved gene clusters encoding the biosynthesis of a polyketide virulence factor in *Burkholderia* spp., and identification of congeners of **1**. A) Genomic alignment. B) Molecular network of **1** and **2** in negative ion mode; shown are the main nodes related to **1**. See the Supporting Information for full network. C) HMBC and COSY correlations of two newly isolated congeners of **1**. D) Chemical relation of **2** to **3** and **1**. LC‐MS monitoring of the formation of **3** (*m*/*z* 387) from **2** (*m*/*z* 387) and of the formation of **1** (*m*/*z* 305) from **2** (*m*/*z* 387); EIC in negative ion mode. E) Model for the formation of **1** from **2**.

To identify congeners of **1** that could potentially be more active than the parent compound, we combined targeted gene inactivation in *B. thailandensis* with a molecular networking approach using the Global Natural Product Social Molecular Networking^15^ (GNPS) platform. We retrieved MS^2^ spectra from extracts of an engineered *bur* overexpression strain (*B. thailandensis* E264 *Pbur*)^14^ and examined the main nodes of the network connected to the *m*/*z* value of **1** (Figure 1 B). Next, we scrutinized a nonproducing mutant (*B. thailandensis* E264 *Pbur*Δ*burJ*) for the loss of the respective *m*/*z* values compared to the overexpression strain. Through this approach we found two chromatographic peaks that correspond to an *m*/*z* of 387 in the negative ion mode in culture extracts from the overexpression strain, while the production of the same metabolites (**2** and **3**) was abolished in our inactivation mutant. Thus, we concluded that **2** and **3** were congeners of **1**. Notably, the production of the more nonpolar congener (**3**) was suppressed when *B. thailandensis* E264 *Pbur* was grown at a constant pH of 6.5 in a bioreactor. By optimization of purification protocols, we succeeded in obtaining both compounds in pure form (**2**, 0.3 mg L^−1^; **3**, 0.4 mg L^−1^).

HRMS data indicated a molecular formula of C_18_H_28_O_7_S for both compounds. Compared to **1** (C_18_H_26_O_4_), **2** and **3** lack one double‐bond equivalent, but are equipped with an additional sulfur atom and three additional oxygen atoms. The predicted sum formula was in full agreement with ^1^H and ^13^C NMR data for both compounds. Through a comparison of the chemical shifts, and COSY and HMBC correlations of **2** with the data for **1**,^14^ it was deduced that **2** showed the same acyl side chain as **1**, consisting of an α,β‐unsaturated Michael acceptor system. HMBC correlations of an ester carbon center, resonating at *δ*=177.8 ppm, to three COSY‐correlated methine protons indicated a γ‐lactone substructure that accounts for one of the two remaining double‐bond equivalents. While **1** has a propionyl side chain, the corresponding spin system was absent in **2**. Furthermore, the ^13^C spectrum of **2** showed two CH_2_ groups shifted to an unusual high field at *δ*=10.5 and 12.1 ppm, typical for cyclopropyl moieties.^16^ The remaining quaternary carbon center (*δ*=54.0 ppm) completed the cyclopropanol substructure, which was linked to the γ‐lactone, according to HMBC correlations.

Based on the molecular formula, we concluded that the thus far elucidated substructure bears an HSO_3_ group, which could either be a sulfite or a sulfonic acid. Owing to the instability of sulfite monoesters, a sulfonic acid would be the more likely candidate. We validated this structure proposal by comparison of the chemical shifts of the respective sulfonate‐substituted carbon atom in **2** and its attached proton with the chemical shifts of model compounds (see Figure S4 in the Supporting Information) all showing highly similar shifts at the respective atoms. Taken together, **2** represents an unprecedented cyclopropanol‐containing sulfonic acid that was named sulfomalleicyprol (Figure 1 C).

In the ^13^C NMR data of the second, less polar compound (**3**) we noticed a shift of the keto moiety resonating at *δ*=196.0 ppm in **2** to a value of *δ*=168.8 in **3**, indicative of an ester moiety. Additionally, we detected a second proton in the α‐position to the lactone carbonyl group, whereas the other parts of **3** showed largely similar shifts compared to the NMR data of **2**. Based on these data, we concluded that the acyl chain was not connected to the lactone ring (as in **2** and **1**), but linked to the cyclopropyl moiety by an ester bond. COSY and HMBC data fully supported the proposed structure of **3**, which was named *iso*‐sulfomalleicyprol (Figure 1 C).

The structures of **2** and **3** suggested that both compounds could be interconverted by an intramolecular reaction. Considering that **3** was not present in cultures that were kept at a slightly acidic pH (6.5) we tested whether **3** could be formed from **2** in vitro under basic conditions, as in cultures growing in shaking flasks (pH 8.2 after 48 h growth in LB medium). By LC‐HRMS monitoring we found that **3** originates from **2** when treated with phosphate buffer (pH 8.1) or K_2_CO_3_ solution (pH 10.6; 5 mm; Figure 1 D). This transformation represents an acyl migration, which most likely proceeds through a retro Claisen condensation with a six‐membered transition state (see Figure S2) formed between the keto and the hydroxy group of **2**. In addition, hydrogen bonding between the sulfonate and the cyclopropanol moiety of **2** is likely to aid in the generation of the required cyclopropanolate for the reaction. Moreover, we detected the formation of **1** when **2** was incubated in aqueous NaOH (pH 14; 5 mm; Figure 1 D). This transformation could be rationalized to proceed through an E1cb mechanism (Figure 1 E) to eliminate the sulfonic acid group with subsequent base‐catalyzed opening of the cyclopropanol ring. These results indicated that **2** is a precursor to both, **1** and **3**. Therefore, we interrogated whether **2** possessed stronger biological activity than its degradation product **1**. When tested with several cell lines, however, **2** did not show elevated cytotoxic/antiproliferative effects compared to **1** (see Table S5).

In light of the similarly low bioactivities of **2** and **1** it appeared plausible that yet another, cryptic precursor could represent the true virulence factor. Therefore, we revisited the extracts of the *bur* overexpression mutant. To exclude the possibility that potential congeners of **1** were overlooked in our molecular network we performed a complementary approach using an all‐ion fragmentation (AIF) experiment in LC‐HRMS with *Pbur* extracts. The AIF data were screened employing the extracted ion chromatogram (EIC) for high‐resolution *m*/*z* values of previously identified MS fragments from **1**, **2**, and **3**. By this approach we identified a parent mass of *m*/*z* 611.3589 (negative ion mode) through its sole dominating fragment ion with *m*/*z* 305.1758 (see Figure S3). Subsequently, we isolated the corresponding compounds as two diastereomers by an optimized purification protocol (**4 a**, 1.8 mg L^−1^, **4 b** 1.5 mg L^−1^). The deduced molecular formula of C_36_H_52_O_8_ from ^13^C and HRMS data for **4** hinted towards a dimeric structure comprising two burkholderic acid‐like subunits (C_18_H_26_O_4_). HMBC and COSY correlations confirmed **4** to be an adduct consisting of two connected γ‐lactones, each linked to an acyl chain. Two additional olefinic carbon atoms resonating at *δ*=157.5 and 132.0 ppm indicated a third α,β‐unsaturated double bond. HMBC correlations to a quaternary carbon center at *δ*=167.9 ppm located this double bond within one of the γ‐lactones, whereas the second γ‐lactone was saturated. The remaining four methylene carbon atoms shifted to high field (*δ*=15.5, 14.8, 12.5, and 9.1 ppm) and two quaternary carbon atoms (*δ*=56.8 and 56.5 ppm) made up two cyclopropanol rings, each connected to one of the γ‐lactones. Thus, we elucidated structure **4** as a dimeric, cyclopropanol‐substituted congener of **1** named bis‐malleicyprol (Figure 2 A). Moreover, we inferred that this dimeric structure resulted from the conjugate addition of the two tautomeric forms, **5** and **6**, of the corresponding monomer. Such a non‐enzymatic addition would rationalize the occurrence of **4** in various stereoisomeric forms. Notably, when incubated under basic conditions, **4 a** isomerizes into **4 b** and other isomeric forms (see Figure S5 A).

**Figure 2:**
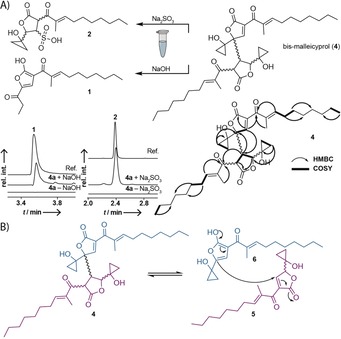
Identification of bis‐malleicyprol (**4**), a dimeric, cyclopropanol‐substituted polyketide linked to the *bur* gene cluster. A) Structure and key COSY and HMBC correlations. Compound **4** can be readily converted into previously isolated products (**1** and **2**) of the *bur* pathway. Also shown is the UHPLC‐MS monitoring of the formation of **1** (*m*/*z* 305.1758) from **4 a**, and of the formation of **2** (*m*/*z* 387.1483) by conjugate addition with Na_2_SO_3_ to **4 a**. EIC in the negative ion mode. B) Chemical equilibrium of **4** with its putative monomers malleicyprol (**5**) and *iso*‐malleicyprol (**6**).

To establish a chemical correlation between the thus far isolated congeners we subjected **4** to various reaction conditions and monitored product formation by LC‐HRMS. Incubation of **4** with Na_2_SO_3_ yielded the previously isolated sulfonic acid **2** (Figure 2 A). Furthermore, we observed the formation of **1** when subjecting **4** to basic conditions. Based on these results, we concluded that the two proposed tautomeric monomers **5**/**6** coexist in a chemical equilibrium with **4** (Figure 2 B). This chemical equilibrium enabled us to trap the reactive species **5** by conjugate addition with Na_2_SO_3_. Thus, we concluded that **4** is a precursor to **2** and **1**. Because of the chemical equilibrium between **5**/**6** and **4** it seems likely that **5** and **6** are the true biosynthetic outcome of the *bur* assembly line, while **4** represents a reversibly formed product of these two highly reactive tautomers. Hence, we named the new compounds malleicyprol (**5**) and *iso*‐malleicyprol (**6**).

The chemical equilibrium between **5**/**6** and **4** allowed us to study the cumulated biological activity of all three species. Cytotoxic and antiproliferative assays with purified **4 a** on various cell lines showed a dramatically increased molar activity, over two orders of magnitude (110‐fold) higher than **1**, originally assigned to the *bur/mal* assembly line (Figure 3 A). Of course, because of the chemical equilibrium between **4** and **5**/**6** the precise contribution of each species to the found biological activity cannot be shown experimentally. Yet, the high reactivity of **5**/**6** suggests that these two tautomers play important roles in the observed toxicity. To validate the effects observed in the whole cell assays, we performed a toxicity assay using the established pathogenicity model, *C. elegans*. As a control, **1** showed no effect on the survival of the nematodes (see Video S1) when added to the growth medium (as high as 100 μg mL^−1^). In stark contrast, when treated with **4 a** (50 μg mL^−1^), no viable nematodes were observed in the corresponding survival assays (Figures 3 C,D; see Video S2). In addition, we performed a *C. elegans* liquid toxicity assay to determine the potency of **4 a** (IC_50_: 0.56 μg mL^−1^; Figure 3 B). Based on these results we propose that **4** and/or **5**/**6** represent the true virulence factors produced by the encoded *bur* assembly line. It is remarkable that the active **6** and inactive **1** differ solely in the C3 (cyclopropanol and propanone) residues (Figure 3 E). Consequently, the reactive cyclopropanol ring of the malleicyprols represents an important pharmacophoric moiety. A prominent example with a similarly strained warhead substructure is the genotoxin colibactin.^17^

**Figure 3:**
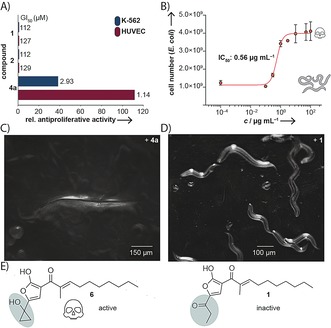
Results from biological activity assays. A) Molar antiproliferative activities of **2** and **4 a** relative to **1**. B) Liquid toxicity assay with *C. elegans* N2 supplemented with **4 a** (bars represent the mean of four replicates ± SD). C) *C. elegans* N2 treated with **4 a** (50 μg mL^−1^). D) *C. elegans* N2 treated with **1** (50 μg mL^−1^). E) Difference in the C_3_ residues of active **6** and inactive **1**.

In conclusion, we have discovered and characterized a new family of structurally intriguing, cyclopropanol‐substituted polyketides. In general, cyclopropanol‐containing natural products are exceedingly rare.^16^, ^18^ We demonstrate that these highly reactive compounds are produced by the *bur/mal* assembly line, which is correlated with virulence in the *B. mallei/pseudomallei* complex, and suggest alternative polyketide virulence determinants. Thus, our results are an important addition to the body of knowledge on small‐molecule disease mediators employed by these infamous human and animal pathogens. This new insight may lead to a better understanding of the molecular basis of glanders and melioidosis, and could facilitate the development of much needed therapeutics^19^ to combat these severe diseases.

## Conflict of interest

The authors declare no conflict of interest.

## Supporting information

As a service to our authors and readers, this journal provides supporting information supplied by the authors. Such materials are peer reviewed and may be re‐organized for online delivery, but are not copy‐edited or typeset. Technical support issues arising from supporting information (other than missing files) should be addressed to the authors.

Supplementary

Supplementary

Supplementary
