# Supplementary material for: Cyclopropanol Warhead in Malleicyprol Confers Virulence of Human‐ and Animal‐Pathogenic Burkholderia Species
Source: Angew Chem Int Ed Engl. 2019 Aug 27;58(40):14129–33. doi: 10.1002/anie.201907324 (PMC6790655; doi:10.1002/anie.201907324)
Supplement: Supplementary file 1 — Supplementary [file ANIE-58-14129-s001.pdf]

Angewandte Chemie
Eine Zeitschrift der Gesellschaft Deutscher Chemiker

Supporting Information

Cyclopropanol Warhead in Malleicyprol Confers Virulence of Human- and Animal-Pathogenic *Burkholderia* Species

*Felix Trottmann, Jakob Franke, Ingrid Richter, Keishi Ishida, Michael Cyrulies, Hans-Martin Dahse, Lars Regestein, and Christian Hertweck**

anie_201907324_sm_miscellaneous_information.pdf
anie_201907324_sm_Video_S1.wmv
anie_201907324_sm_Video_S2.wmv

Content

Experimental Procedures **7**

Bacterial Strains and General Culture Conditions 5

Nematode Strains and Maintenance 7

Bioreactor Conditions 7

Preparation of *B. thailandensis PburΔburJ* Mutant 6

General Analytical Procedures 8

Mass Spectral Network analysis 9

Isolation of Sulfomalleicyprol and iso-Sulfomalleicyprol 9

Isolation of bis-Malleicyprol 12

Synthesis of γ-Butyrolactone-3-sulfonic acid 15

Synthesis of Sulfomalleicyprol (**2**) from bis-Malleicyprol (**4**) 15

Transformations of Sulfomalleicyprol (**2**) 15

Transformation of bis-Malleicyprol (**4**) to Burkholderic Acid (**1**) 15

Antiproliferative and cytotoxic activites of **1**, **2** and **4** 16

Nematode Toxicity Assays 15

Supporting References **40**

Supplementary Figures

Supplementary Figure 1. Mass spectral network **5**

Supplementary Figure 2. Six-membered transition state **5**

Supplementary Figure 3. All Ion Fragmentation **6**

Supplementary Figure 4. ^{13}C and ^{1}H NMR data at the sulfonate substituted carbon **5**

Supplementary Figure 5. Additional information to main Figure 2A **6**

Supplementary Figure 6. *B. thailandensis* PburΔ*bur*J PCR Product **8**

Supplementary Figure 7. Key ^{1}H-^{1}H COSY and HMBC correlations of **2** **10**

Supplementary Figure 8. Key ^{1}H-^{1}H COSY and HMBC correlations of **3** **11**

Supplementary Figure 9. Key COSY and HMBC correlations of **4** **13**

Supplementary Figure 10. Key HMBC and COSY correlations γ-butyrolactone-3-sulfonic acid**15**

Supplementary Figure 11. IR spectrum of **2** **18**

Supplementary Figure 12. IR spectrum of **3** **18**

Supplementary Figure 13. IR spectrum of **4** **19**

Supplementary Figure 14. IR spectrum of γ-butyrolactone-3-sulfonic acid **18**

Supplementary Figure 15. ^{1}H NMR spectrum of **2** (CD_3CN) **19**

Supplementary Figure 16. ^{13}C NMR spectrum of **2** (CD_3CN) **20**
Supplementary Figure 17. DEPT135 spectrum of **2** (CD_3CN) **21**
Supplementary Figure 18. COSY spectrum of **2** (CD_3CN) **21**
Supplementary Figure 19. HSQC spectrum of **2** (CD_3CN) **22**
Supplementary Figure 20. HMBC spectrum of **2** (CD_3CN) **22**
Supplementary Figure 21. ^{1}H NMR spectrum of **2** (CD_3OD) **23**
Supplementary Figure 22. ^{13}C NMR spectrum of **2** (CD_3OD) **23**
Supplementary Figure 23. DEPT135 spectrum of **2** (CD_3OD) **24**
Supplementary Figure 24. COSY spectrum of **2** (CD_3OD) **24**
Supplementary Figure 25. HSQC spectrum of **2** (CD_3OD) **25**
Supplementary Figure 26. HMBC spectrum of **2** (CD_3OD) **25**
Supplementary Figure 27. ^{1}H NMR spectrum of **3** (CD_3OD) **26**
Supplementary Figure 28. ^{13}C NMR spectrum of **3** (CD_3OD) **26**
Supplementary Figure 29. DEPT135 spectrum of **3** (CD_3OD) **27**
Supplementary Figure 30. COSY spectrum of **3** (CD_3OD) **27**
Supplementary Figure 31. HSQC spectrum of **3** (CD_3OD) **28**
Supplementary Figure 32. HMBC spectrum of **3** (CD_3OD) **28**
Supplementary Figure 33. ^{1}H NMR spectrum of **4a** (CD_3OD) **29**
Supplementary Figure 34. ^{13}C NMR spectrum of **4a** (CD_3OD) **29**
Supplementary Figure 35. DEPT135 spectrum of **4a** (CD_3OD) **30**
Supplementary Figure 36. COSY spectrum of **4a** (CD_3OD) **30**
Supplementary Figure 37. HSQC spectrum of **4a** (CD_3OD) **31**
Supplementary Figure 38. HMBC spectrum of **4a** (CD_3OD) **31**
Supplementary Figure 39. ^{1}H NMR spectrum of **4a** ($CDCl_3$) **32**
Supplementary Figure 40. ^{13}C NMR spectrum of **4a** ($CDCl_3$) **32**
Supplementary Figure 41. DEPT135 spectrum of **4a** ($CDCl_3$) **33**
Supplementary Figure 42. COSY spectrum of **4a** ($CDCl_3$) **33**
Supplementary Figure 43. HSQC spectrum of **4a** ($CDCl_3$) **34**
Supplementary Figure 44. HMBC spectrum of **4a** ($CDCl_3$) **33**
Supplementary Figure 45. ^{1}H NMR spectrum of **4b** ($CDCl_3$) **34**
Supplementary Figure 46. ^{13}C NMR spectrum of **4b** ($CDCl_3$) **34**
Supplementary Figure 47. DEPT135 spectrum of **4b** ($CDCl_3$) **35**
Supplementary Figure 48. COSY spectrum of **4b** ($CDCl_3$) **35**
Supplementary Figure 49. HSQC spectrum of **4b** ($CDCl_3$) **36**
Supplementary Figure 50. HMBC spectrum of **4b** ($CDCl_3$) **36**
Supplementary Figure 51. ^{1}H NMR spectrum of γ-butyrolactone-3-sulfonic acid (D_2O) **38**
Supplementary Figure 52. ^{13}C NMR spectrum of γ-butyrolactone-3-sulfonic acid (D_2O) **38**

Supplementary Figure 53. COSY spectrum of γ-butyrolactone-3-sulfonic acid (D_2O) **39**

Supplementary Figure 54. HSQC spectrum of γ-butyrolactone-3-sulfonic acid (D_2O) **39**

Supplementary Figure 55. HMBC spectrum of γ-butyrolactone-3-sulfonic acid (D_2O) **40**

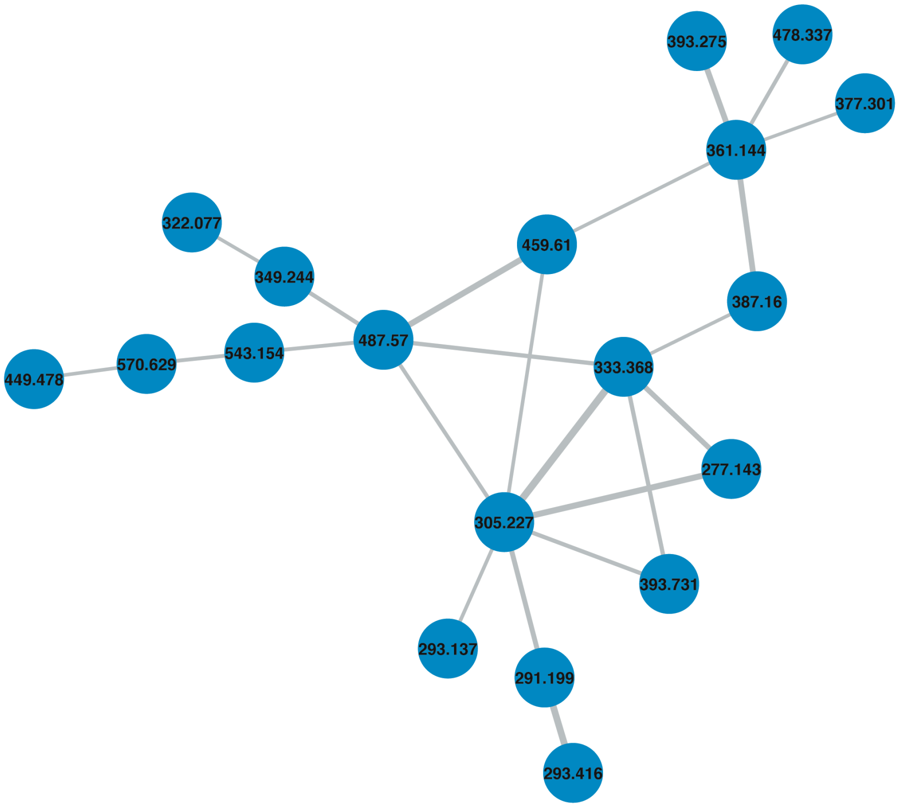

Supplementary Figure 1. Mass spectral network in negative ion mode including burkholderic acid (*m*/*z* 305.2; [M–H]$^-$; **1**) and sulfomalleicyprol (*m*/*z* 387.2; [M–H]$^-$; **2**) with line strength indicating cosine score (see below).

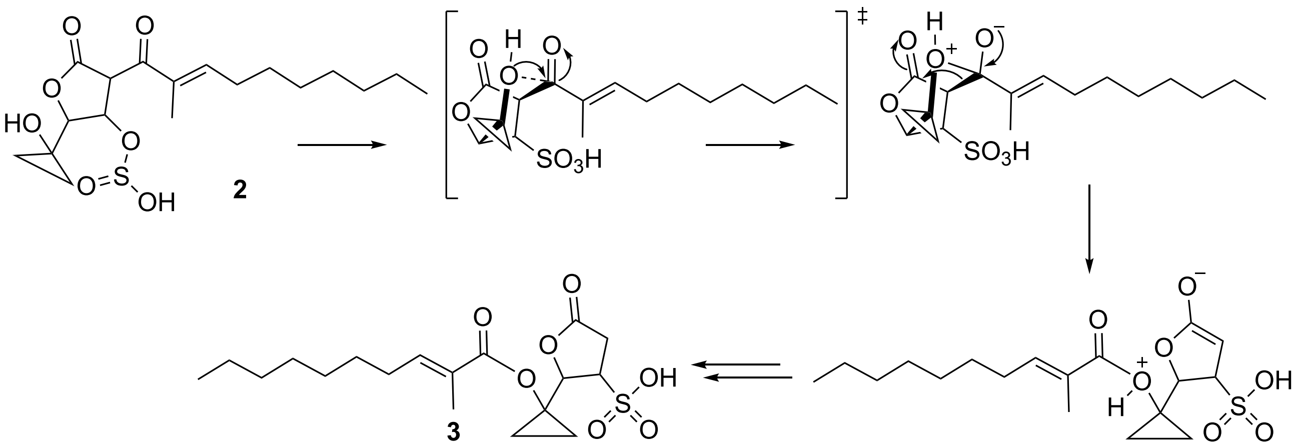

Supplementary Figure 2. Proposed model for the rearrangement of **2** to **3** via a six-membered transition state. Similar intramolecular retro-Claisen reaction from the synthetic literature have been hypothesized to proceed via six[1]- or seven[2]-membered intermediates.

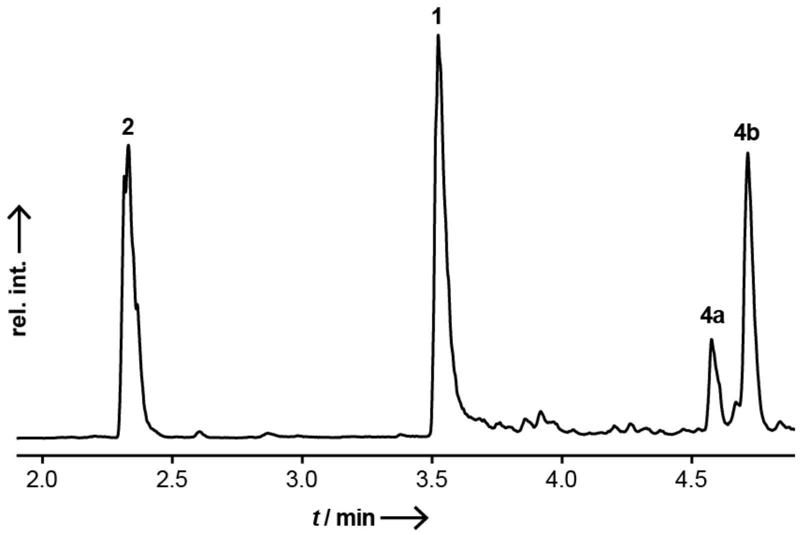

Supplementary Figure 3. All Ion Fragmentation (AIF) profile, EIC in negative ion mode for *m/z* 305.1758 ± 0.5 ppm. **1** represents non-fragmented burkholderic acid (**1**); **2** represents a major fragment ion formed from sulfomolleicyprol (**2**); **4** represents the dominating fragment (*m/z* 305.1758) ion formed from bis-malleicyprol **(4)**.

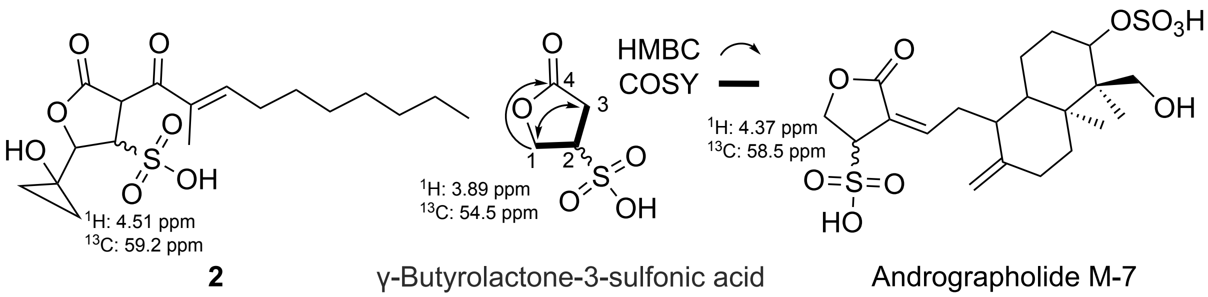

Supplementary Figure 4. Comparison of the ^{13}C and ^{1}H NMR data at the sulfonate substituted carbon in **2**, γ-butyrolactone-3-sulfonic acid (see below for synthesis and spectra, Supplementary Figure 10) and the Andrographolide Metabolite 7.[3]

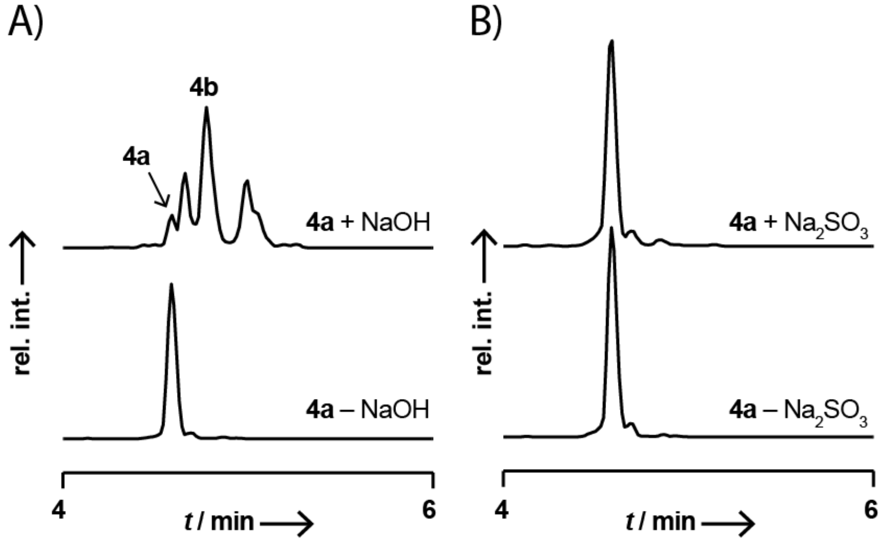

Supplementary Figure 5. Additional information to main Figure 2A: UHPLC-MS traces for **4a** **(**extracted ion chromatogram for *m/z* 611.3589 in negative ion mode)**;** A) Assay for the transformation of **4a** to **1**. **B)** Assay for the conjugate addition of Na_2SO_3 to **4a**.

Experimental Procedures

Bacterial Strains and General Culture Conditions

Burkholderia thailandensis wild-type strain E264 was obtained from the DSMZ GmbH (Braunschweig). *B. thailandensis* E264 was cultured in LB liquid medium or agar at 30 °C. For mutant strains, tetracycline (45 µg mL^{-1}) and/or kanamycin 150 µg mL^{-1}) were used as selection marker.

Nematode Strains and Maintenance

Bacterial strains (*E.coli* OP50) were cultured in Lysogeny broth (10 g L^{-1} tryptone, 5 g L^{-1} yeast extract, 10 g L^{-1} NaCL, pH 7.0) at 37 °C overnight. Bacteria were harvested by centrifugation at 8000 g for 5 min, washed twice in sterile K-medium (3.1 g L^{-1} NaCl, 2.4 g L^{-1} KCl) and resuspended in K-medium to an OD_{600} of approximately 1.2. before use.
Caenorhabditis elegans wild-type N2 (var. Bristol) was obtained from the *C. elegans* Geneteics Centre (CGC, University of Minnesota, USA). Stocks were maintained by transferring 1 cm^2 pieces of nematode growth medium (NGM[4]) agar with nematodes onto fresh plates of NGM seeded with *E. coli* OP50. *C. elegans* was cultured at 20 °C for 5–7 days. *C. elegans* wild-type N2 was harvested by washing a large petri dish with 12 mL K-medium. The suspension was incubated at 4 °C for 20 min. The supernatant was removed and the suspension (5 mL) containing nematode worms stored at 4 °C until needed. The number of worms could not be accurately standardized without the use of specialist worm counting machinery.[5] However, mixing the worm suspension prior to use allowed a similar number of worms to be used in one experiment.

Bioreactor Conditions

All shake flask experiments were performed in 1 L Erlenmeyer flasks with a shaking frequency of 150 rpm, a shaking diameter of 25 mm and a filling volume of 200 mL. The scale up criterion in the 4 L stirred tank reactor (Sartorius) was the oxygen transfer rate.[6] The filling volume of the reactor system was 1 L. The pH was controlled at a value of 6.5 by adding 25% NH_4OH. To ensure aerobic conditions, the dissolved oxygen tension was controlled by the stirring rate at a minimum value of 20% and a constant aeration of 1 vvm. The initial glucose concentration was 10 g L^{-1}. The system was inoculated with initial OD600 of 0.1.

Preparation of *B. thailandensis PburΔburJ* Mutant

A genomic DNA isolation from *B. thailandensis* E264 was carried out by Wizard® Genomic DNA Purification Kit (Promega). A gene fragment containing *burJ* (ABC34346, putative acyl CoA ligase gene) was amplified by PCR with the primers II2096fw (5'-GTG TCC TCG CTT TCA CGA CAA TTG-3') and II2096rv (5'-TCA TGC GGA TTC CCG CTC GC-3') using 5prime PCR Extender Polymerase (VWR international). The amplicon was purified with

GFX™ PCR DNA and Gel Band Purification Kit (GE Healthcare). The PCR product containing the kanamycin (Kan) resistance gene, which was amplified from pK19[7] with the primers Km1200fw (5'-CCT GCG TGC AAT CCA TCT TGT TC-3') and Km1200rv (5'-TTG GTC GGT CAT TTC GAA CC-3') using 5prime PCR Extender Polymerase, was purified using the above-mentioned procedure. The amplicon was cloned into pGEM T-easy vector (Promega) and the resulting plasmid was restricted with *Not*I and blunted using the Klenow fragment (NEB). This blunted kanamycin resistance cassette gene was cloned into the *Sgr*AI restricted plasmid, which was SAP (Shrimp Alkaline Phosphatease, NEB) treated, generating pGEM-Δ*burJ*. This plasmid was restricted with *Not*I and the obtained Δ*burJ* gene fragment was cloned into pGL42a[8] which was restricted with *Not*I and SAP treated, generating pGL42a-Δ*burJ*.
B. thailandensis E264 *Pbur* was pre-cultured overnight at 30 °C. Overnight cultured cells were inoculated in LB medium (1/100 dilution) and cultured up to an OD_{600} between 0.4 and 0.8 at 30 °C. The cultured broth was centrifuged and the supernatant was removed. Precipitated cells were resuspended in sucrose solution (300 mM) and centrifuged. After repeating this washing step twice, the washed cells were resuspended in 300 mM sucrose and subjected to electroporation (200 kV) with deletion plasmids (1−10 µg). Transformed cells were precultured in LB broth (1 mL) for 4−6 h to overnight at 30 °C with shaking and then plated on either LB or MM9[9] with 2 mg mL^{-1} 4-chloro phenylalanine agar plates with tetracycline (45 µg mL^{-1}) and kanamycin (150 µg mL^{-1}). After 2 to 4 days, a few positive colonies were observed and confirmed by colony PCR (Supplementary Figure 5).

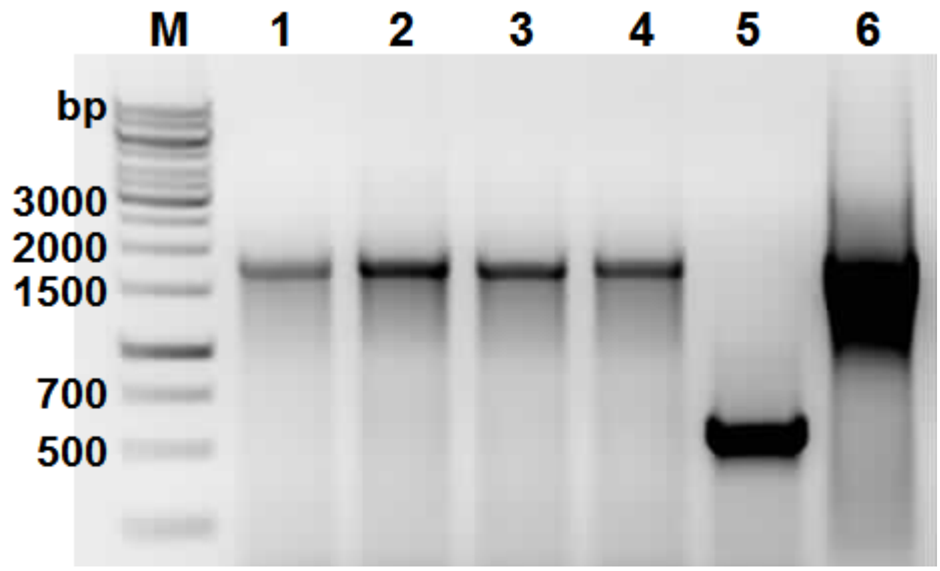

Supplementary Figure 6. *PCR amplification with the DNA from B. thailandensis Pbur*ΔburJ *(lanes 1−4), Wild type (lane 5), and pGL42a-Δ*burJ *(lane 6), M: marker. Estimated size of amplicons, using the primer pair II2096fw2 (5'-CCG ATC AAC CCG GCG CTC AC-3') and II2096rv2 (5'-AAC GCG GAC AGG AAC GCC GG-3'): mutant: 1718 bp (lanes 1–4, 6), Wild type: 559 bp (lane 5).*

General Analytical Procedures

NMR spectra were measured on a Bruker Avance III 600 MHz spectrometer with cryo probe in CD_3OD, $CDCl_3$, CD_3CN and D_2O. Spectra were referenced relative to the residual solvent peak (CD_3OD: δ_H = 3.30, δ_C = 49.0 ppm; $CDCl_3$: δ_H = 7.28, δ_C = 77.0 ppm; CD_3CN: δ_H = 1.94, δ_C = 1.3; 118.3 ppm; D_2O: δ_H = 4.70).

LC-HRMS measurements were carried out on an UltiMate 3000 UHPLC (Thermo Fisher Scientific) coupled to a Thermo Fisher Scientific QExactive HF-X Hybrid Quadrupole-Orbitrap with an electrospray ion source using a Kinetex 100-1.7 C_{18} column (50 × 2.1 mm, Phenomenex) and an elution gradient [solvent A: H_2O + 0.1% HCOOH, solvent B: acetonitrile, 5% to 100% B in 4.5 min, 100% B for 2 min, 100% B to 5% B in 0.001 min, 5% B for 1.5 min; flow rate: 0.7 mL min^{-1}, injection volume: 2 µL]. $HRMS^2$ measurements and All Ion Fragmentation (AIF) experiments were carried out on the same system operating in Parallel Reaction Monitoring (PRM) or AIF mode, respectively.

For LCMS measurements a Hewlett Packard Agilent 1100 MSD using an Eclipse XDB C_8 column (150 × 4.6 mm; Agilent) and an elution gradient [solvent A: H_2O + 0.1% HCOOH, solvent B: acetonitrile, gradient: 20% B for 5 min, 20% to 99% B in 30 min, 99% B for 1 min, 99% B to 20% B in 1 min, 20% B for 7 min, flow rate: 1 mL min^{-1}, injection volume: 20 µL], with DAD, an electrospray ion source and a High Capacity Ion Trap (HCT, Bruker) mass analyzer were used. LC-MS-MS spectra for networks were recorded for a number of 3 precursor ions at once in Auto MS^2 mode with negative polarity and a precursor selection range from *m*/*z* 100–600.

For IR measurements, a FTIR4100 (Jasco) with ATR technique was used. Manual baseline correction and smoothing was applied to all IR spectra.

Mass Spectral Network Analysis

LC-MS^2 spectra obtained from ethyl acetate extracts of *B. thailandensis* E264 *Pbur* were uploaded to the Global Natural Products Social Molecular Networking server (GNPS, http://gnps.ucsd.edu). Settings for analysis were as follows: precursor ion mass tolerance: 1.0 Da; fragment ion mass tolerance: 0.5 Da; minimum pair cosine: 0.3; minimum matched peaks: 3; the remaining parameters were used at their standard values. Subsequently, the network data was visualized and manually cropped with Cytoscape 3.3.0.

For manual cropping, nodes that resulted from fewer than 5 spectra and showed low abundance (lower than 1.0E6) were removed. In addition, networks that consisted of less than 4 nodes in total were omitted from analysis. The remaining two main networks were displayed with continuous mapping with regards to the cosine score (a linear gradient of 0 to 10.8 points between cosine 0.0 and 1.0). The edge-weighted spring embedded layout was used. For further analysis, only the network that showed a node representing burkholderic acid (*m*/*z* 305) was considered. The *m*/*z* values from the respective nodes were searched via their extracted ion chromatogram in extracts of the overexpression mutant *B. thailandensis* E264 *Pbur* and a gene inactivation mutant. (*B. thailandensis* E264 *Pbur*Δ*burJ*).

Isolation of Sulfomalleicyprol (2) and *iso*-Sulfomalleicyprol (3)

B. thailandensis E264 *Pbur* was grown in 1 L baffled Erlenmeyer flasks filled with 300 mL of MM9[9] medium supplemented with 45 mg L^{-1} tetracycline for 48 h at 30 °C with shaking at 150 rpm. Subsequently, the cultures were extracted twice with ethyl acetate and concentrated under reduced pressure. The crude extract was subjected to silica chromatography with a

mixture of chloroform/methanol as solvent and a stepwise increasing proportion of methanol (from 4% to 100% MeOH). Fractions containing the target molecules were pooled, concentrated and further purified by preparative HPLC with a Nucleosil C_{18} column (100-7, 250 × 21, Macherey-Nagel) [solvent A: H_2O + 0.1% TFA, solvent B: acetonitrile 83%, gradient: 40% B to 70% B in 40 min, flow rate: 15 mL min^{-1}] to obtain sulfomalleicyprol at 0.3 mg L^{-1} and *iso*-sulfomalleicyprol at 0.4 mg L^{-1}.

Sulfomalleicyprol (2). NMR data see Table S1; **IR** (ATR): $\tilde{\nu}(T_{rel})$ 3519 (br w), 3336 (br w), 2922 (m), 2858 (m), 1770 (m), 1674 (s), 1539 (w), 1457 (w), 1193 (s), 1143 (s), 1049 (s), 841 (w), 807 (w), 721 (w), 639 (w) cm^{-1}; **HRMS** (ESI): *m*/*z* calc. for $C_{18}H_{27}O_7S$ $(M–H)^-$ 387.1483, found 387.1479 (1.0 ppm).

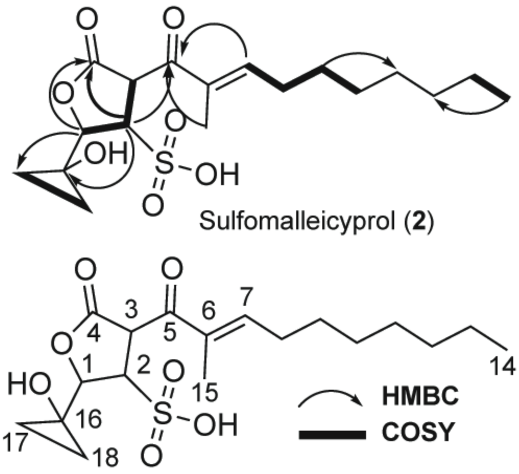

Supplementary Figure 7. Key ^1H-^1H COSY and HMBC correlations of **2**.

Supplementary Table 1. NMR Data of **2**.

Sulfomalleicyprol (**2**)				
	CD_3CN (600 MHz, 298 K)		CD_3OD (600 MHz, 298 K)	
**Position**	δ_C	δ_H (*J* [Hz],m)	δ_C	δ_H (*J* [Hz],m)
1	85.7	4.41 (6.0, d)	86.2	4.35 (5.9; d)
2	61.4	4.08 (6.0; 7.1, dd)	59.2	4.51 (5.9; d)
3	52.3	4.77 (7.1, d)	n.d	
4	172.8		171.8	
5	195.7		193.0	
6	136.7		135.5	
7	149.6	7.05 (7.5, t)	148.4	7.07 (m)
8	29–33	2.33 (m)	29.1	2.38 (m)
9	29.1	1.51 (7.6, qi)	28.1	1.57 (7.7; qi)
10	29–33	1.33 (m)	29.1	1.42 (m)
11	29–33	1.33 (m)	31.6	1.34 (m)
12	29–33	1.33 (m)	22.4	1.34 (m)
13	23.4	1.33 (m)	29.0	1.34 (m)
14	14.4	0.89 (7.0, t)	13.1	0.93 (6.8; t)
15	12.5	1.79 (s)	10.6	1.85 (s)
16	56.3		54.0	
17	11.8	0.80 (m)	10.5	0.90 (m)
18	11.7	0.80 (m)	12.1	0.90 (m)

***iso*-Sulfomalleicyprol (3). NMR** data see Table 2; **IR** (ATR): $\tilde{\nu}(T_{rel})$ 3452 (br m), 2925 (m), 2857 (m), 1780 (s), 1716 (s), 1645 (w), 1,457 (w), 1341 (w), 1176 (s), 1040 (s), 731 (w), cm^{-1}; **HRMS** (ESI): *m*/*z* calc. for $C_{18}H_{27}O_7S$ $(M–H)^-$ 387.1483, found 387.1488 (1.3 ppm).

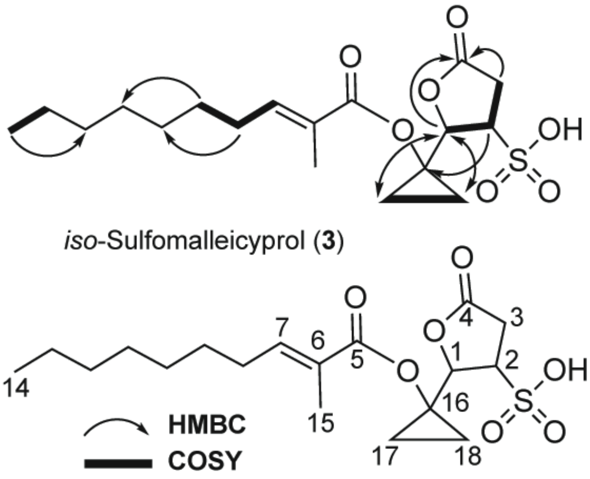

Supplementary Figure 8. Key ^1H-^1H COSY and HMBC correlations of **3**.

Supplementary Table 2. NMR Data of **3**.

iso-Sulfomalleicyprol (**3**)		
CD_3OD (600 MHz, 298 K)		
**Position**	δ_C	δ_H (*J* [Hz],m)
1	84.7	4.81 (2.4, d)
2	58.0	3.90 (m)
3	32.0	2.83 (m)
4	177.2	
5	168.8	
6	128.5	
7	145.2	6.75 (m)
8	29.7	2.19 (7.5, q)
9	29.6	1.44 (m)
10	30–33	1.32 (m)
11	30–33	1.32 (m)
12	30–33	1.32 (m)
13	23.7	1.32 (m)
14	14.4	0.90 (m)
15	12.4	1.78 (m)
16	60.8	
17	10–12	1.09 (m)
18	10–12	1.09 (m)

Isolation of bis-Malleicyprol (4)

Cultures of *B. thailandensis* E264 Pbur were grown in 1 L baffled Erlenmeyer flasks filled with 200 mL of MM9 (in total 4 L) or LB (in total 4 L) medium (each containing tetracycline at 45 mg L^{-1}) medium at 30 °C and 150 rpm for 48 h. Cultures in LB medium were extracted three times with ethyl acetate and concentrated under reduced pressure; cultures grown in MM9 medium were extracted with XAD16. The XAD16 resin was washed with water and subsequently eluted with ethyl acetate followed by elution with methanol. Both eluted fractions were combined and concentrated under reduced pressure. The obtained extracts (either from cultures grown in LB or MM9) were separately subjected to silica chromatography. In both instances, a mixture of chloroform/methanol was used as solvent with a stepwise increasing proportion of methanol (from 4% to 100% MeOH). Subsequently, all fractions containing the target molecule (as judged by LC-MS, mixture of diasteromers **4a** and **4b**) were pooled, concentrated under reduced pressure and dissolved in 60 mL water. The aqueous pool was extracted twice with dichloromethane and the pooled organic extracts concentrated under reduced pressure. This step was followed by repeated rounds of purification via preparative HPLC, first using a Nucleosil 100-7 C_{18} column (250 × 20 mm, Macherey-Nagel) [solvent A: H_2O + 0.1% TFA, solvent B: acetonitrile 83%, gradient: 70% B for 5 min followed by 70% B to 100% B in 30 min then 100% B for 15 min, flow rate: 15 mL

min^{-1}] and then a Nucleodur HTec 110-5 C_{18} column (250 × 10 mm, Macherey-Nagel) [solvent A: H_2O + 0.1% TFA, solvent B: acetonitrile, 84% B for 30 min flow rate: 6 mL min^{-1}] to obtain 1.8 mg L^{-1} pure compound **4a** and 1.5 mg L^{-1} of compound **4b**. Compound **4b** showed greater instability than **4a** with a tendency to degrade to compound **4a** (including other non-isolatable isomers and degradation products; data not shown).

bis-Malleicyprol (4). NMR data see Table S3; **IR** (ATR): $\tilde{\nu}(T_{rel})$ 3391 (br w), 2925 (m), 2858 (m), 1768 (s), 1647 (s), 1458 (w), 1245 (m), 1175 (m), 1029 (m), 900 (w), 802 (w), 718 (w) cm^{-1}; **HRMS** (ESI): *m/z* calc. for $C_{36}H_{51}O_8$ $(M–H)^-$ 611.3589, found 611.3579 (1.6 ppm).

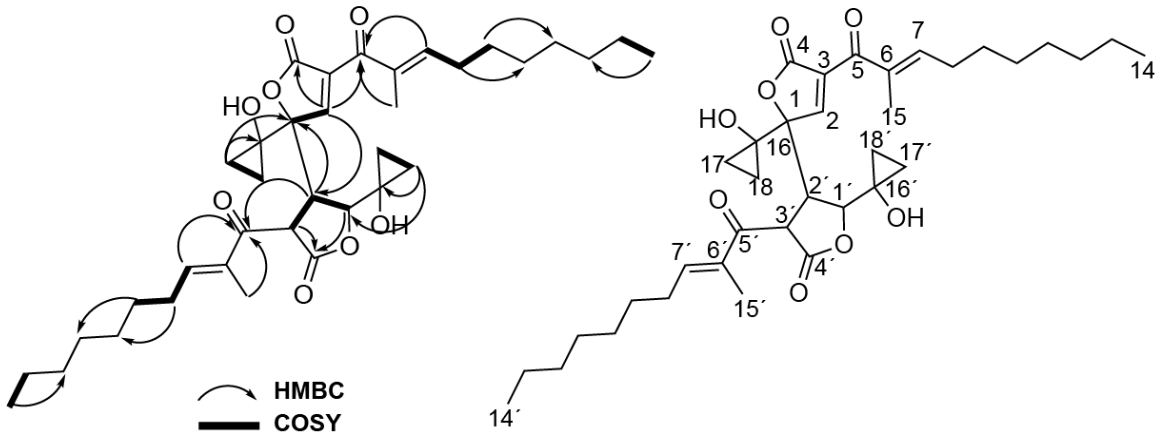

bis-Malleocyprol (**4**)

Supplementary Figure 9. Key COSY and HMBC correlations of **4**.

Supplementary Table 3. NMR data of **4**: diastereomers **4a** and **4b**

bis-malleicyprol (**4**)

	**4a**				**4b**	
	$CDCl_3$ (600 MHz, 298 K)		CD_3OD (600 MHz, 298 K)		$CDCl_3$ (600 MHz, 298 K)	
**Pos.**	δ_C	δ_H (*J* [Hz],m)	δ_C	δ_H (*J* [Hz],m)	δ_C	δ_H (*J* [Hz],m)
1	85.8		89.6		84.3	
2	157.5	7.51 (s)	158.0	7.88 (s)	158.9	7.92 (s)
3	132.0		134.7		131.7	
4	167.9		169.6		167.9	
5	188.4		191.2		189.6	
6	137.0		138.2		137.1	
7	149.9	6.43 (7.2, t)	152.1	6.57 (m)	151.1	6.58 (7.2, t)
8	29-32	2.28 (m)	29.5	2.34 (m)	29-30	2.35 (m)
9	28.40	1.45 (7.0, qi)	30-33	1.49 (m)	28.4	1.48 (m)
10	29-32	1.3 (m)	30-33	1.4–1.3 (m)	29-30	1.3 (m)
11	29-32	1.3 (m)	30-33	1.4–1.3 (m)	29-30	1.3 (m)
12	29-32	1.3 (m)	30-33	1.4–1.3 (m)	29-30	1.3 (m)
13	22.6	1.3 (m)	23.7	1.4–1.3 (m)	22.6	1.3 (m)
14	14.1	0.9 (m)	14.4	0.89 (m)	13.8	0.91 (m)
15	11.0	1.84 (s)	11.0	1.84 (s)	11-12	1.90 (s)
16	56.8		58.8		58.2	
17	9.1	0.33 (m); 0.82 (m)	10.9	0.51 (m); 0.64 (m)	7.4	0.30 (m); 0.72 (m)
18	14.8	1.04 (m); 1.16 (m)	12-14	0.85 (m)	14-15	0.45 (m); 0.81 (m)
1′	82.5	4.51 (7.7, d)	85.9	4.21 (7.3, d)	83.4	3.61 (8.5, d)
2′	43.0	4.58 (8.7, t)	45.0	4.43 (7.4, d)	40.3	4.82 (m)
3′	48.6	4.21 (9.4, d)	n.d.		50.4	4.83 (m)
4′	169.7		173.0		169.2	
5′	192.9		194.9		194.6	
6′	136.0		137.5		136.1	
7′	150.1	6.73 (7.1, t)	149.0	6.89 (m)	151.7	6.99 (7.1, t)
8′	29.8	2.34 (m)	29.6	2.34 (m)	29-30	2.45 (m)
9′	28.3	1.52 (7.2, qi)	30-33	1.54 (m)	28.4	1.59 (m)
10′	29-32	1.3 (m)	30-33	1.4–1.3 (m)	29-30	1.3 (m)
11′	29-32	1.3 (m)	30-33	1.4–1.3 (m)	29-30	1.3 (m)
12′	29-32	1.3 (m)	30-33	1.4–1.3 (m)	29-30	1.3 (m)
13′	22.6	1.3 (m)	23.7	1.4–1.3 (m)	22.6	1.3 (m)
14′	14.1	0.9 (m)	14.4	0.89 (m)	13.8	0.91 (m)
15′	11.8	1.84 (s)	12.0	1.82 (s)	11-12	1.93 (s)
16′	56.5		55.7		54.3	
17′	12.4	1.08 (m); 0.96 (m)	12–14	0.85 (m)	14-15	1.0 (m); 0.81 (m)
18′	15.5	1.05 (m); 1.17 (m)	12–14	0.85 (m)	14-15	1.0 (m); 0.72 (m)

Synthesis of γ-Butyrolactone-3-sulfonic acid

γ-Butyrolactone-3-sulfonic acid was prepared following a procedure by Yllner[10]. The obtained crude product was further purified through preparative HPLC with a Synergi Hydro-RP C_{18} column (80-10, 250 × 21.2 mm, Phenomenex) [solvent A: H_2O, solvent B: acetonitrile 83%, 95% B for 10 min, flow rate: 15 mL min^{-1}].

γ-Butyrolactone-3-sulfonic acid. NMR data see Table S4; **IR** (ATR): $\tilde{\nu}(T_{rel})$ 3618 (w), 3550 (w), 1765 (s), 1620 (m), 1156 (s), 1038 (s), 997 (s), 849 (w), 724 (s) cm^{-1}; **HRMS** (ESI): *m/z* calc. for $C_4H_5O_5S$ $(M–H)^-$ 164.9863, found 164.9850 (7.8 ppm).

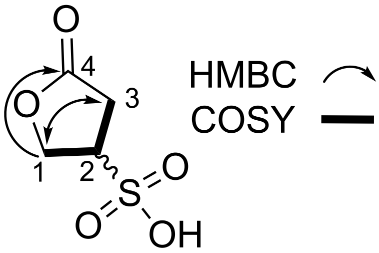

Supplementary Figure 10. Key HMBC and COSY correlations γ-butyrolactone-3-sulfonic acid.

Supplementary Table 4. NMR data of γ-butyrolactone-3-sulfonic acid

γ-butyrolactone-3-sulfonic acid		
D_2O (500 MHz, 298 K)		
**Position**	δ_C	δ_H (*J* [Hz],m)
1	69.8	4.60 (m)
2	54.5	3.89 (m)
3	30.8	H^a: 3.05 (9.6, 18.7, dd)<br>H^b: 2.80 (3.7, 18.7, dd)
4	179.0	

Synthesis of Sulfomalleicyprol (2) from bis-Malleicyprol (4)

In the insert of an HPLC vial, 100 µg (163.2 nmol) of **4a** were dissolved in 9 µL DMSO and 1 µL of an aqueous Na_2SO_3 solution (1 eq; 20.6 mg mL^{-1}) was added. The resulting solution was incubated for 15 min in a sonication bath. Subsequently, 1 µL of the solution was diluted with 99 µL MeOH and directly analyzed via UHPLC-HRMS^2.

Transformation of Sulfomalleicyprol (2) to *iso*-Sulfomalleicyprol (3) and Burkholderic Acid (1)

For transformations to burkholderic acid, 47.5 µg of **2** were dissolved in 100 µL NaOH (5 mM) while for transformations to *iso*-sulfomalleicyprol, 47.5 µg of **2** were dissolved in100 µL

K_2CO_3 (5 mM). The resulting solutions were incubated at 37 °C for 100 min, diluted 1:100 with MeOH and directly analyzed through UHPLC-HRMS[2].

Transformation of bis-Malleicyprol (4) to Burkholderic Acid (1)

47.5 µg of **4a** were dissolved in 9 µL DMSO and 1 µL of aqueous NaOH (40 mg mL^{-1}) was added. The previously clear solution immediately turned bright yellow. From this solution, 1 µL was diluted with 99 µL of MeOH and directly analyzed via UHPLC-HRMS[2].

Antiproliferative and Cytotoxic Activities of 1, 2 and 4a

Cell assays were conducted with human umbilical vein endothelial cells HUVEC (ATCC CRL-1730) and human chronic myeloid leukemia cells K-562 (DSM ACC 10) for antiproliferative effects and with human cervix carcinoma cells HeLa (DSM ACC 57) for cytotoxic effects as previously described.[11] Values for burkholderic acid as previously reported.[9]

Supplementary Table 5. Biological acitivites of **1**, **2** and **4a**.

**Compound**	**Antiproliferative effect [µM]**		**Cytotoxicity [µM]**
	HUVEC GI_{50}	K-562 GI_{50}	HeLa CC_{50}
**1**	127	112	67
**2**	129	112	68
**4a**	1.14	2.93	24.78

Nematode Toxicity Assays

Plate-based toxicity assays were performed in 24-well plates (Costar, Corning, NY, USA) containing 1.5 mL NGM agar seeded with 30 µL of *E. coli* OP50 form an overnight culture. The bacterial lawn was allowed to grow at room temperature for 2 days. For exposure to chemicals, a 30 µL drop of each chemical at the appropriate concentration (or solvent control), sufficient to cover the entire surface of the well, was dried on the agar at room temperature for 30 min in a laminar flow hood. Each chemical concentration was tested in at least three replicate wells. Boric acid dissolved in water (0–18 mM) was used as a positive control[4]. Concentrations of **1** and **4a** (dissolved in methanol) tested in the agar assay were 0.6, 3, and 50 µg mL^{-1}.

Nematode worms were washed from NGM plates as described above. The resulting nematode worm suspension (10 µL) was added to the center of each well and the plates were dried at room temperature for 30 min in a laminar flow hood. Plates were incubated at 20 °C and an image of each individual well was captured daily using a Zeiss Axio Zoom.V16 Stereomicroscope (Zeiss, Oberkochen, Germany) until the bacterial lawn was completely consumed by the worms.[12]

To calculate chemical exposure concentration, the volume of the agar was taken into account. For example, dropping 30 µL of 900 mM boric acid onto 1.5 mL NGM agar resulted in a final concentration of 18 mM boric acid during exposure of nematode worms.

Liquid experiments were conducted in six-well cell culture plates (Costar). Aliquots of 1.7 mL *E. coli* suspension (OD_{600} of 1.2) were placed into each well. In liquid toxicity experiments 100 µL of **4a** (final concentration 0.1–100 µg mL^{-1}) were added to the wells (4 replicates). A blank control consisted of 100 µL methanol, while a toxicity control consisted of 100 µL 360 mM boric acid (final concentration of 18 mM).
Nematode worms were washed from NGM plates as described above and 200 µL of gently vortexed nematode suspension was added to three wells. Each plate also contained 3 wells without added nematodes (200 µL *E. coli* suspension, OD_{600} of 1.2). These allow the natural degradation of the bacteria in K-medium with and without chemicals to be measured. The plates were incubated at 20 °C and 50 rpm for 5 days.
Since the bacterial cell number is directly related to the number of viable nematode worms in the suspension, the bacterial cell number (*E. coli* OP50) was measured at 24 h intervals using a CASY® cell counter Model TT (Roche Innovatis AG, Bielefeld, Germany). Cell numbers measured at day 5 were used to determine IC50 values and 95% confidence intervals (CI) from four-parameter sigmoidal concentration-dependent response curves using GraphPad Prism Version 5.03 (GraphPad Software, La Jolla, California, USA, www.graphpad.com).

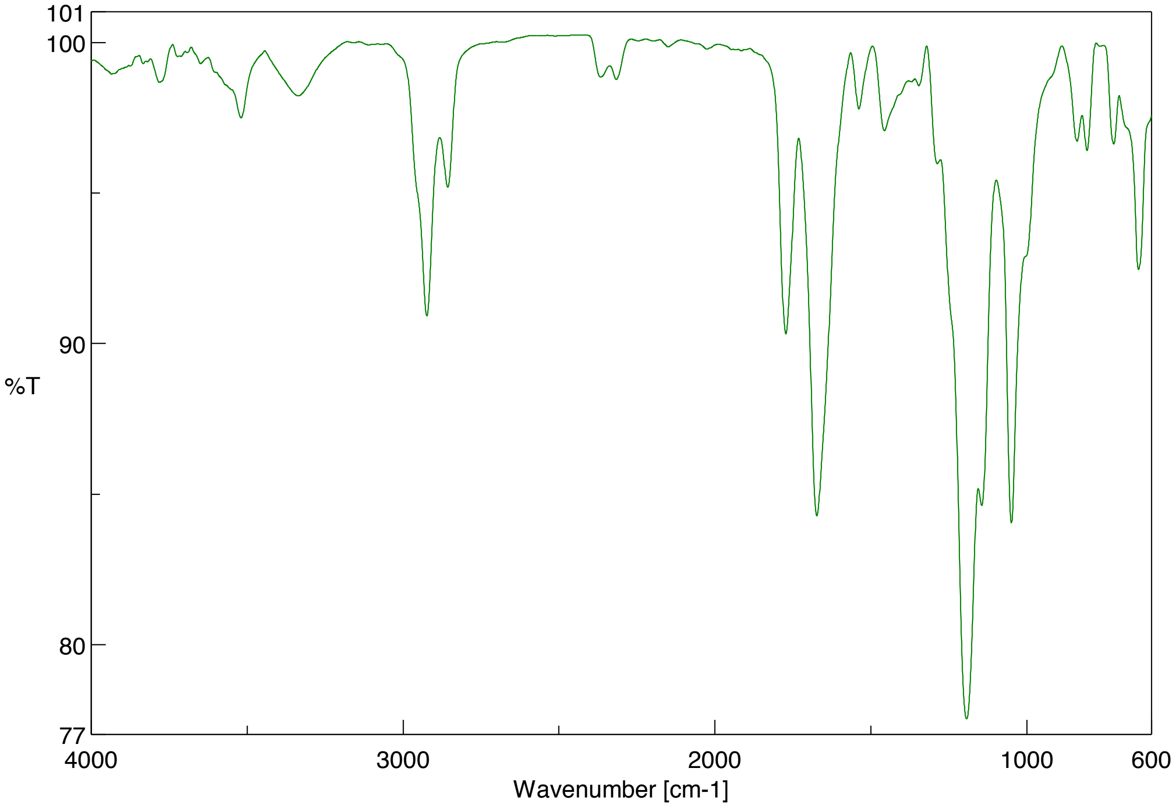

Supplementary Figure 11. IR spectrum of **2**.

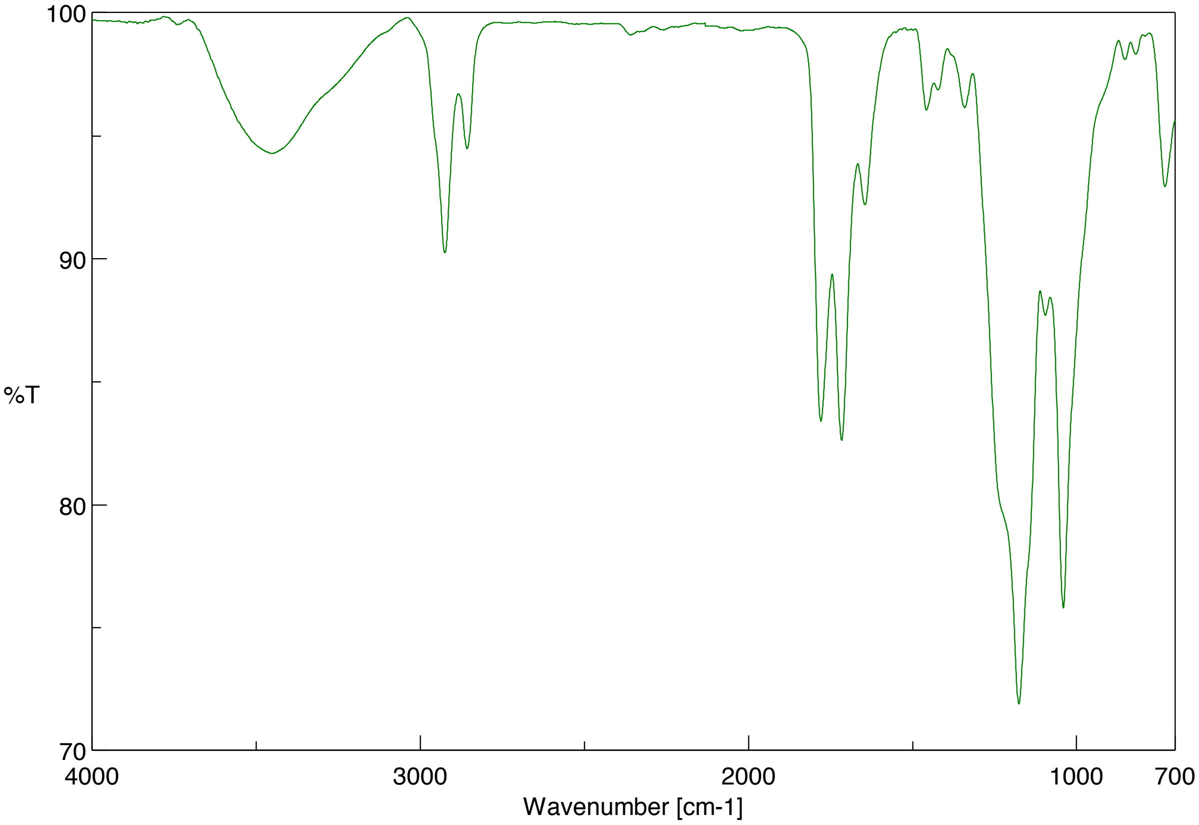

Supplementary Figure 12. IR spectrum of **3**.

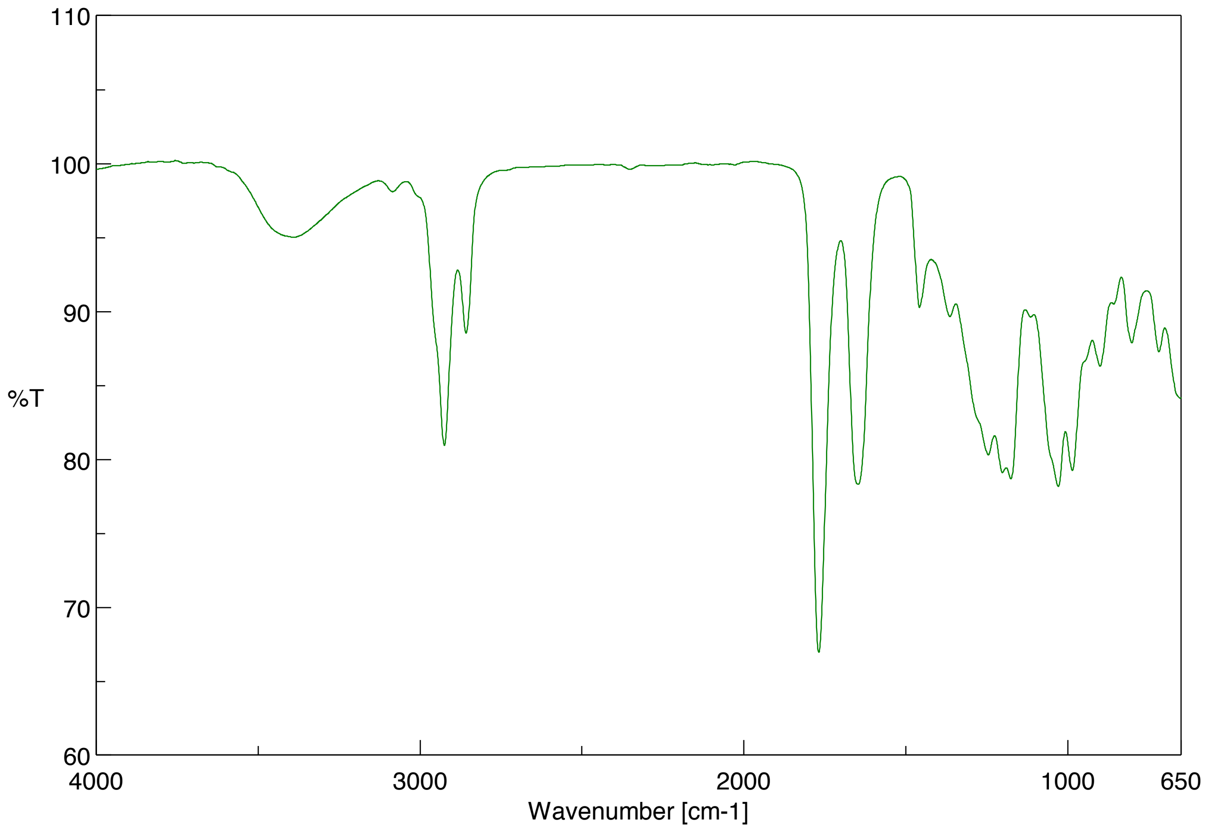

Supplementary Figure 13. IR spectrum of **4a**.

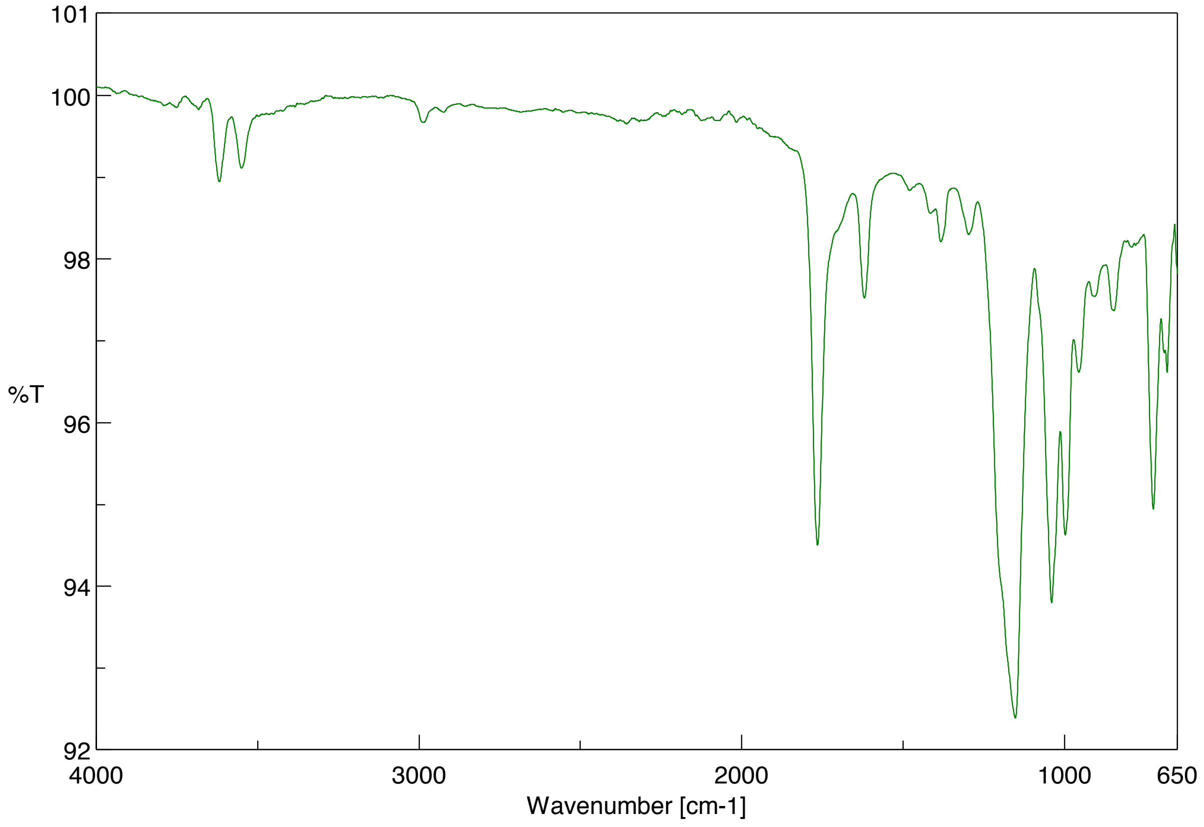

Supplementary Figure 14. IR spectrum of *γ-Butyrolactone-3-sulfonic acid*.

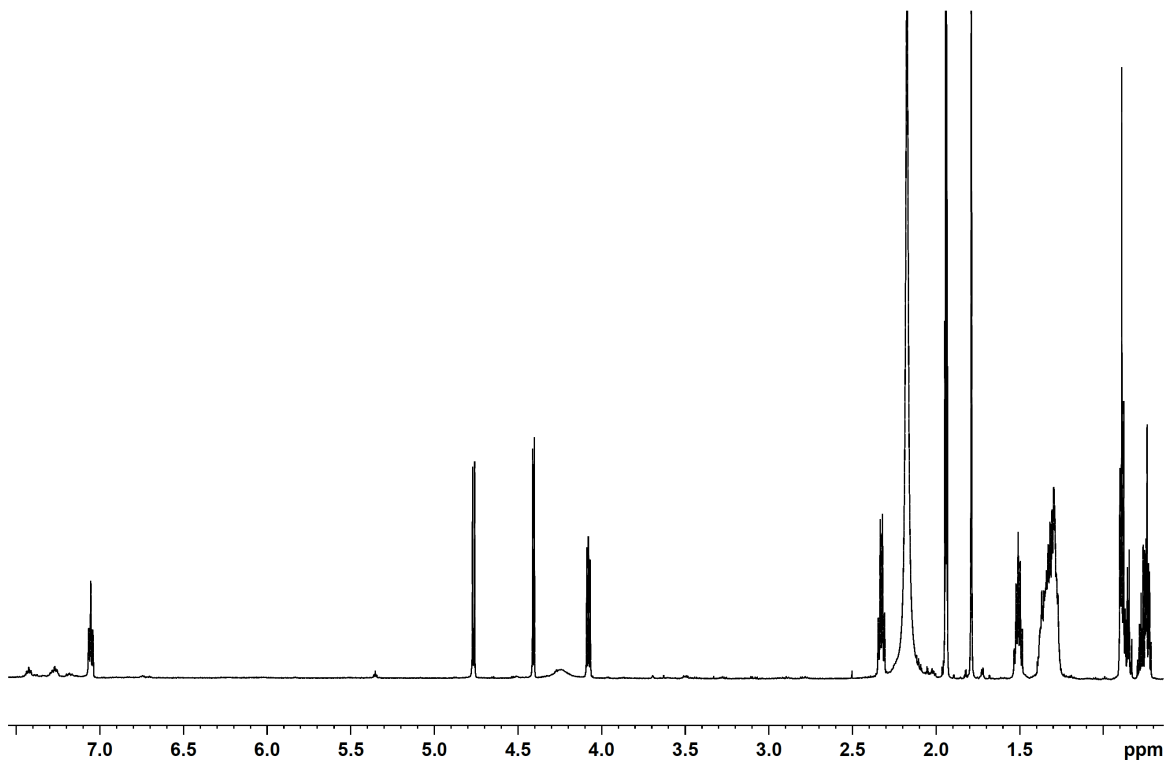

Supplementary Figure 15. ^{1}H NMR spectrum of **3** (CD_3CN).

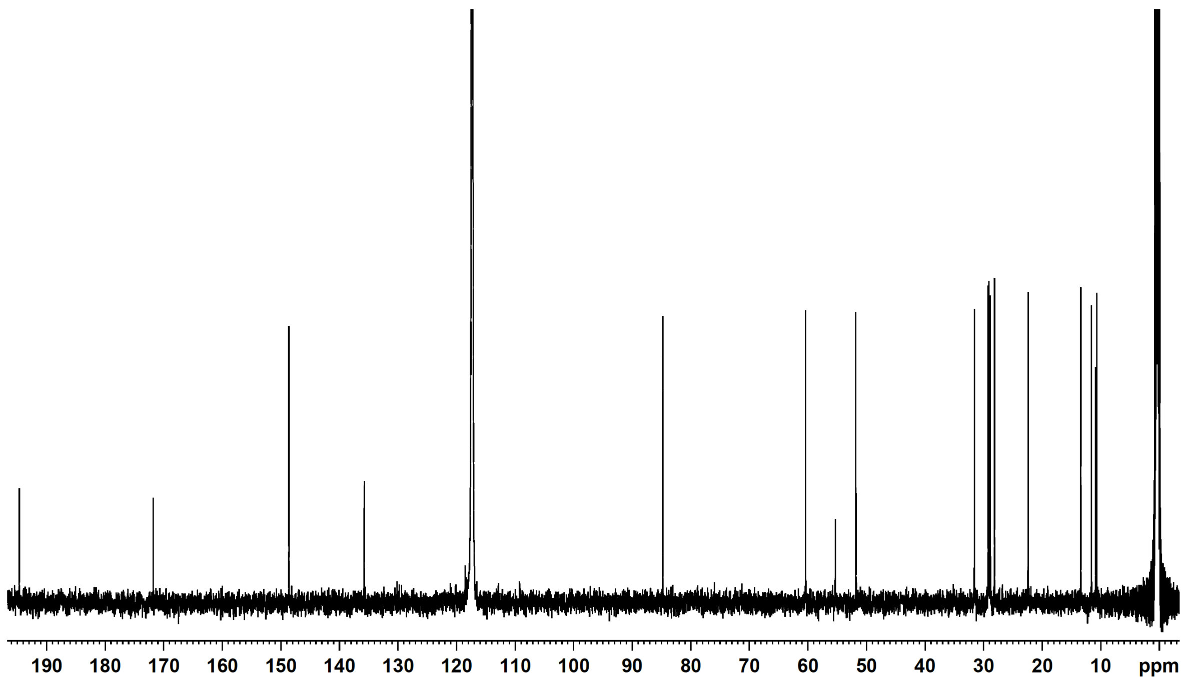

Supplementary Figure 16. ^{13}C NMR spectrum of **2** (CD_3CN).

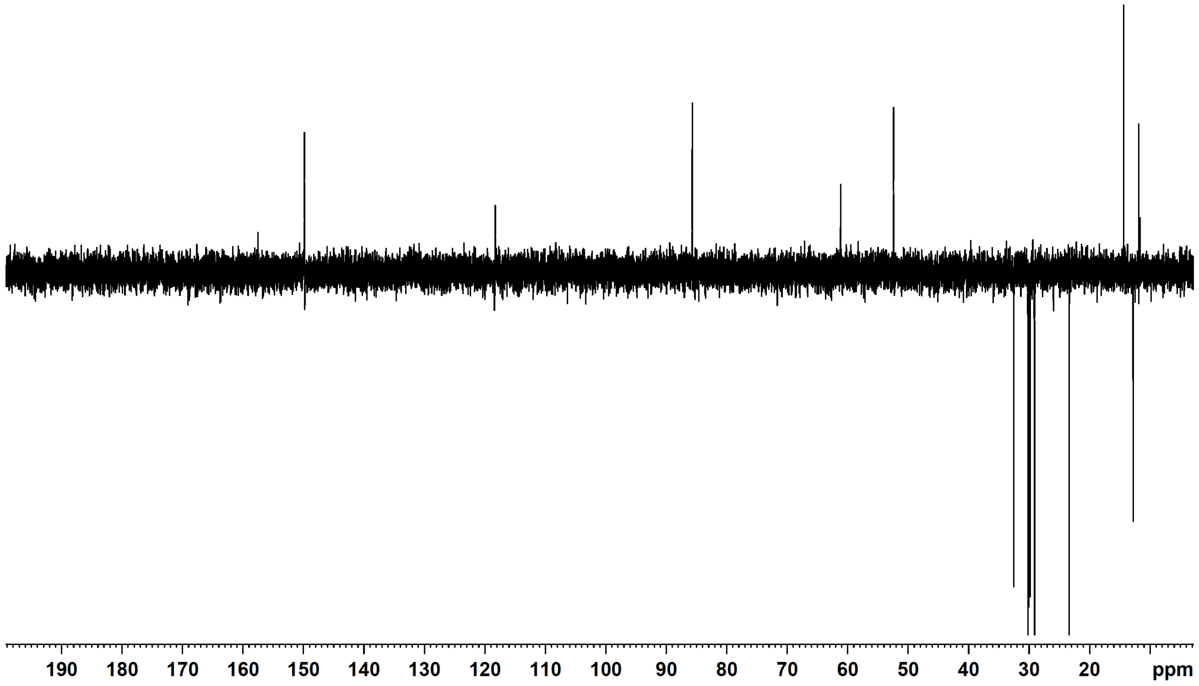

Supplementary Figure 17. DEPT135 spectrum of **2** (CD_3CN).

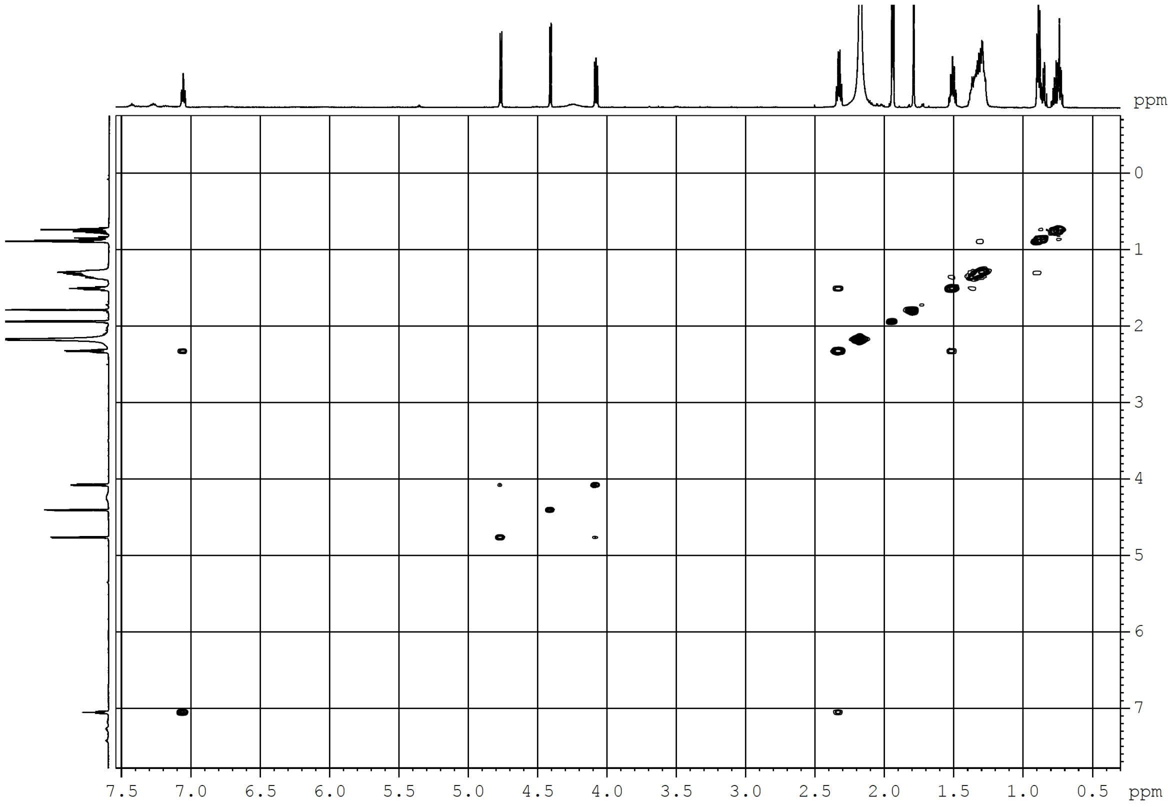

Supplementary Figure 18. COSY spectrum of **2** (CD_3CN).

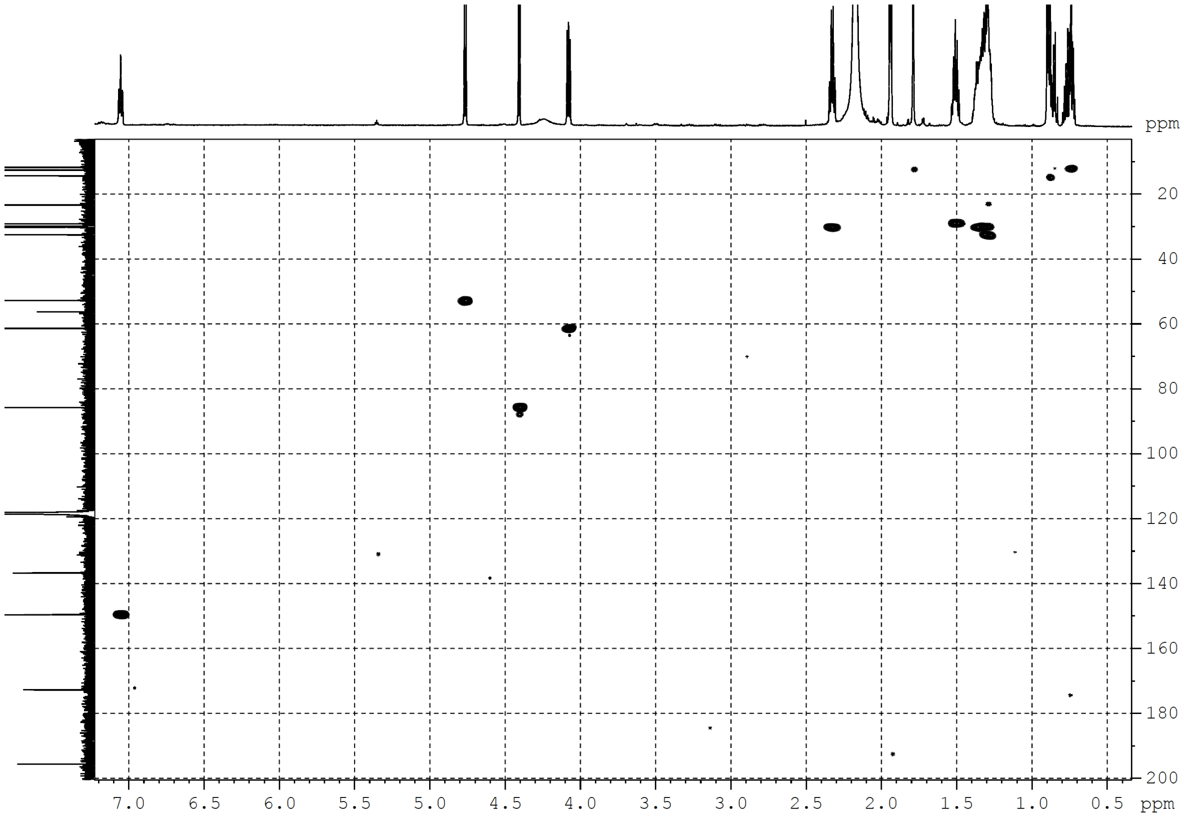

Supplementary Figure 19. HSQC spectrum of **2** (CD_3CN).

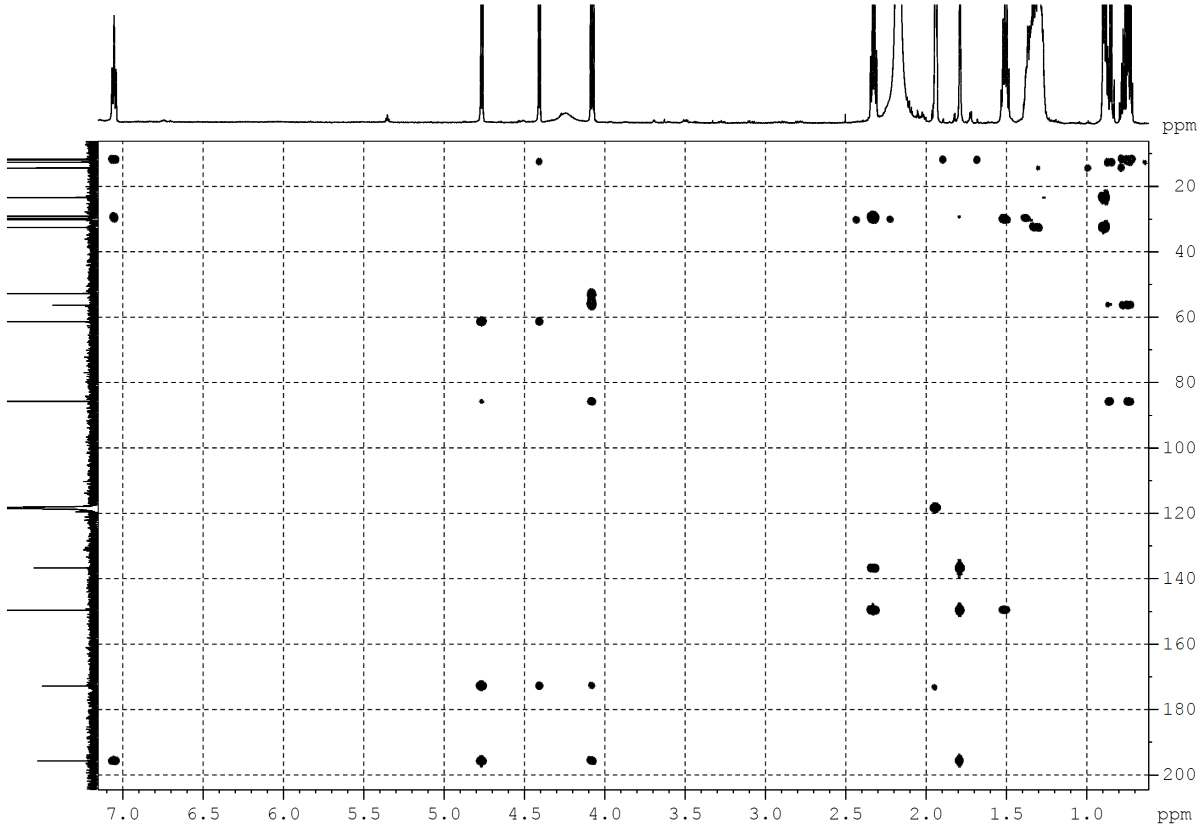

Supplementary Figure 20. HMBC spectrum of **2** (CD_3CN).

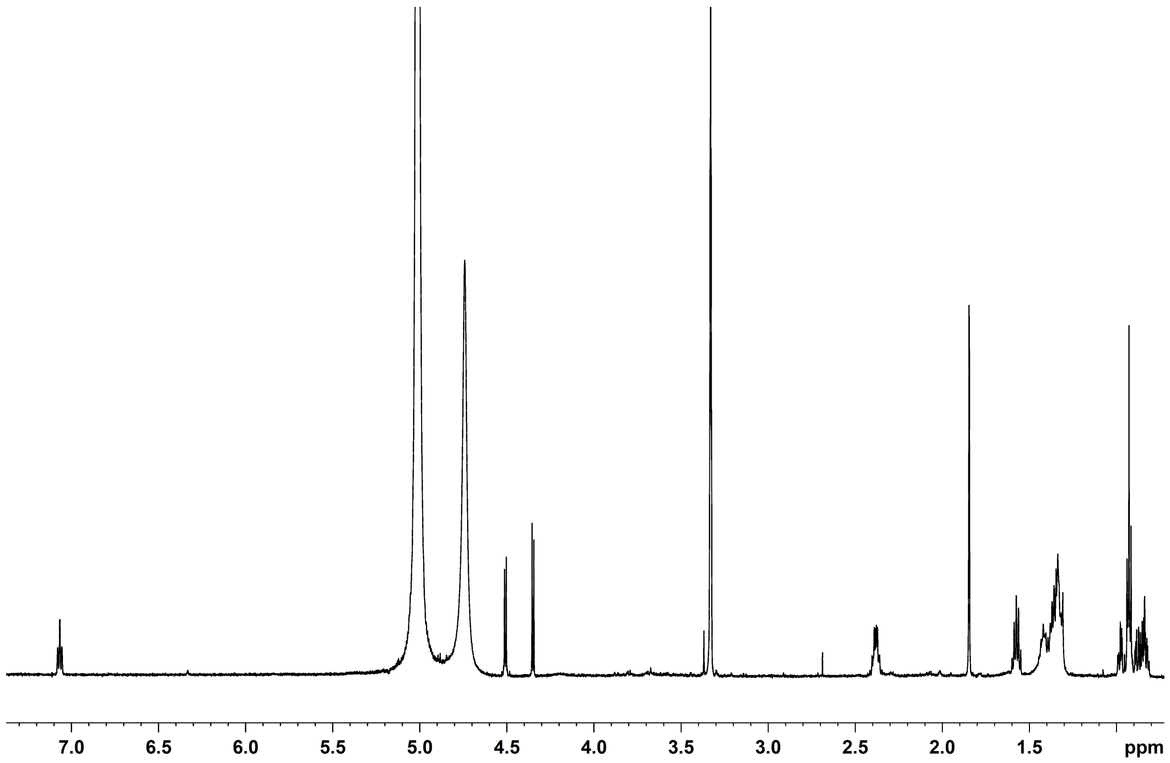

Supplementary Figure 21. ^{1}H NMR spectrum of **2** (CD_3OD).

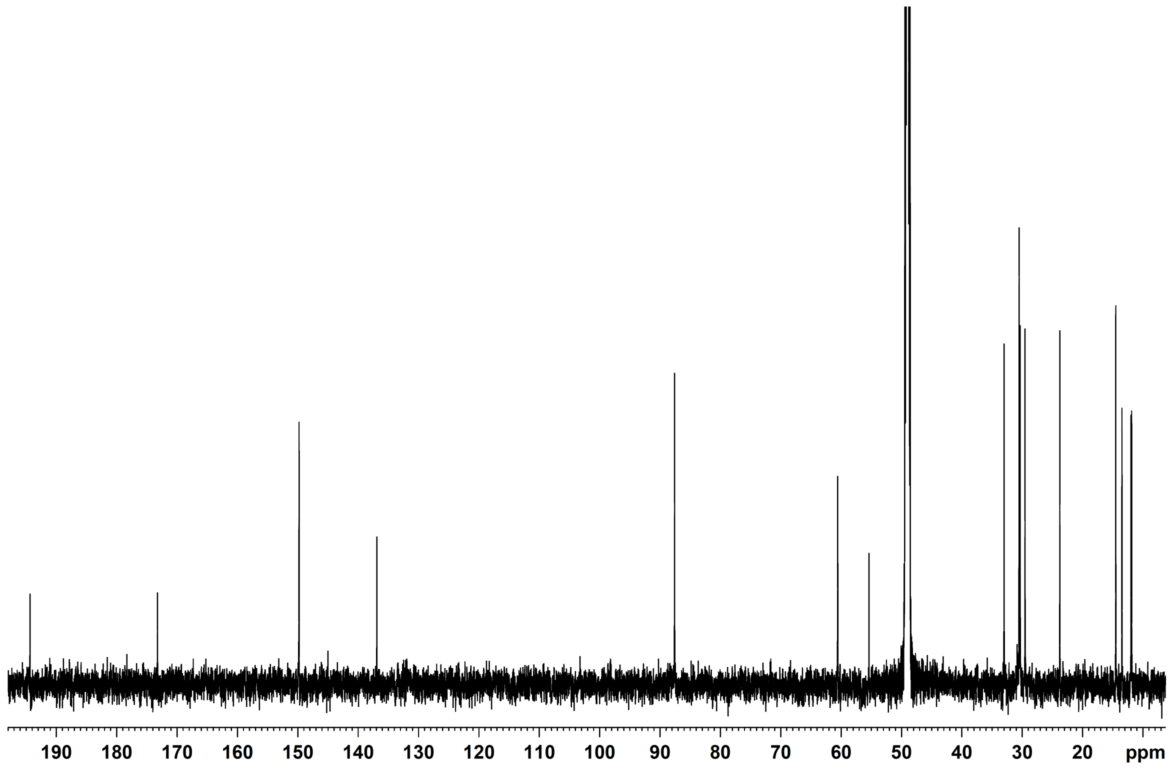

Supplementary Figure 22. ^{13}C NMR spectrum of **2** (CD_3OD).

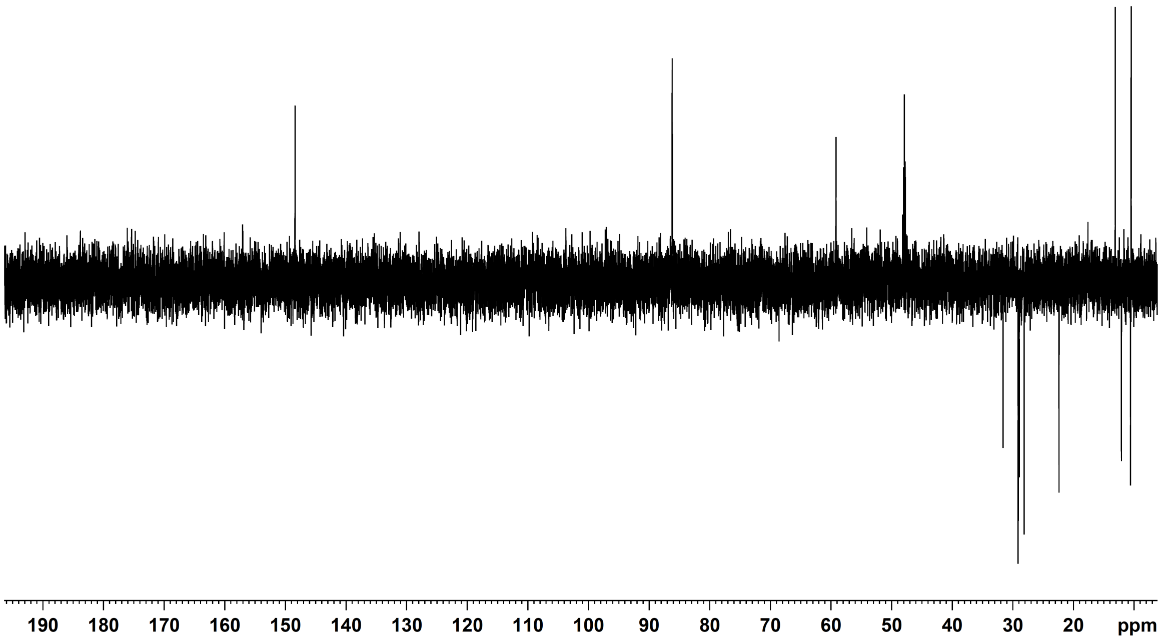

Supplementary Figure 23. DEPT135 spectrum of **2** (CD_3OD).

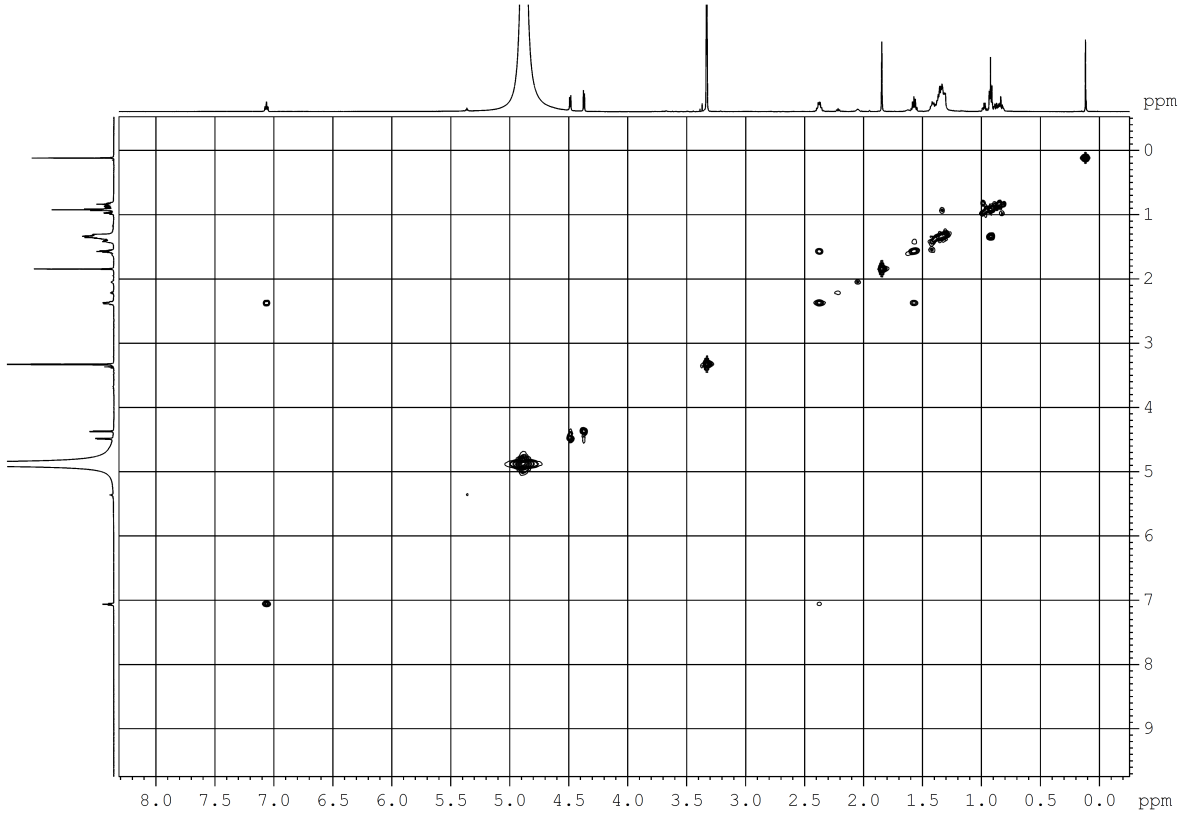

Supplementary Figure 24. COSY spectrum of **2** (CD_3OD).

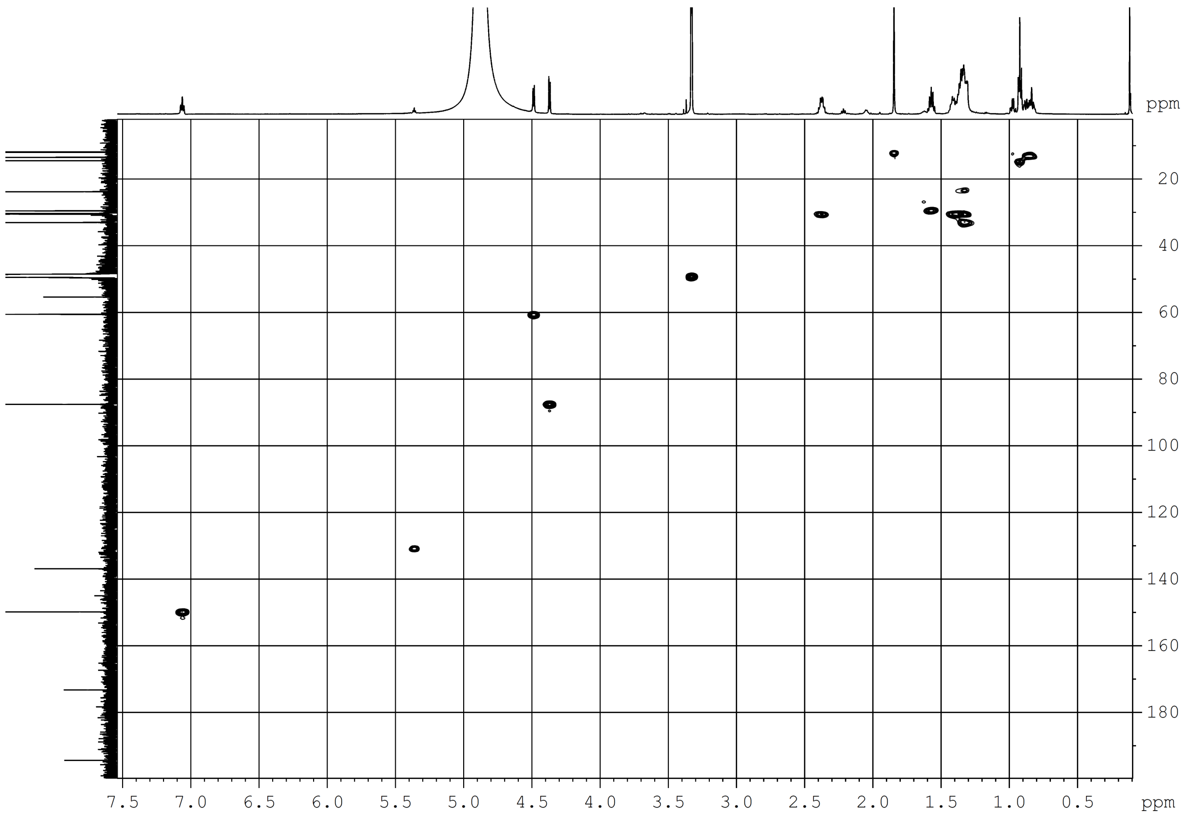

Supplementary Figure 25. HSQC spectrum of **2** (CD_3OD).

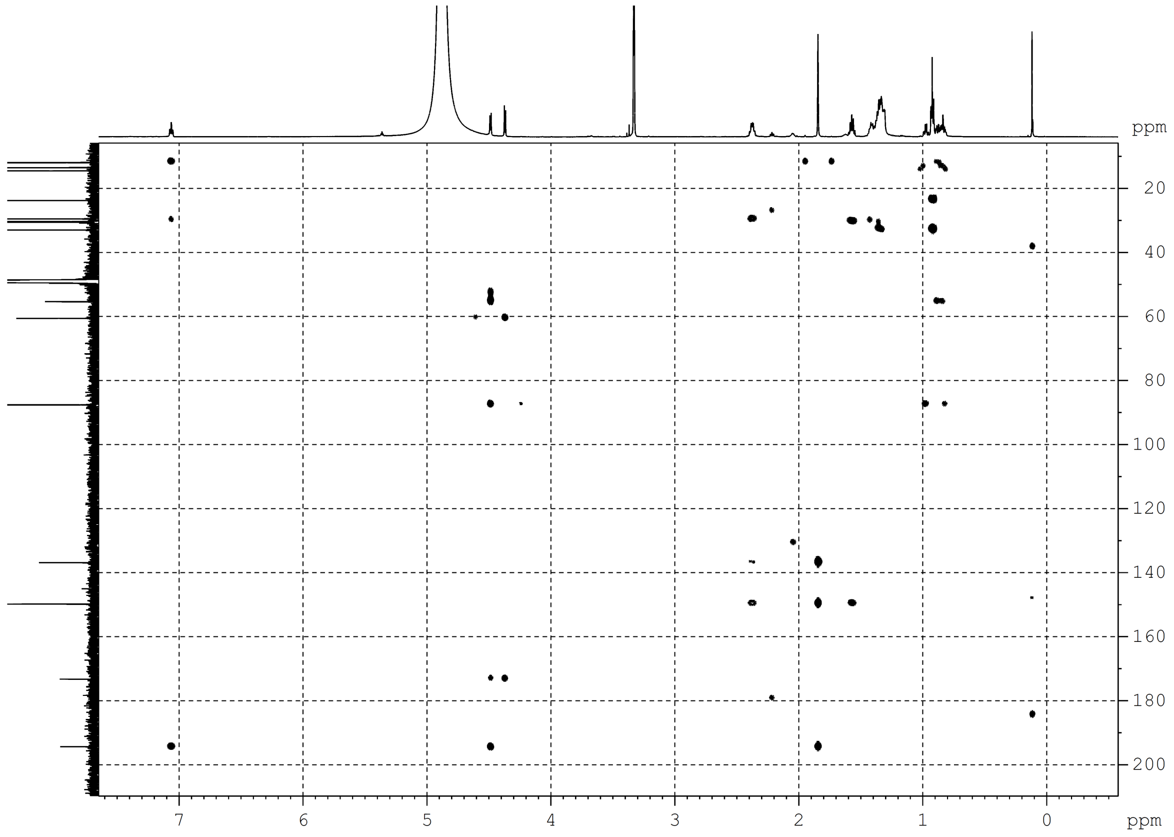

Supplementary Figure 26. HMBC spectrum of **2** (CD_3OD).

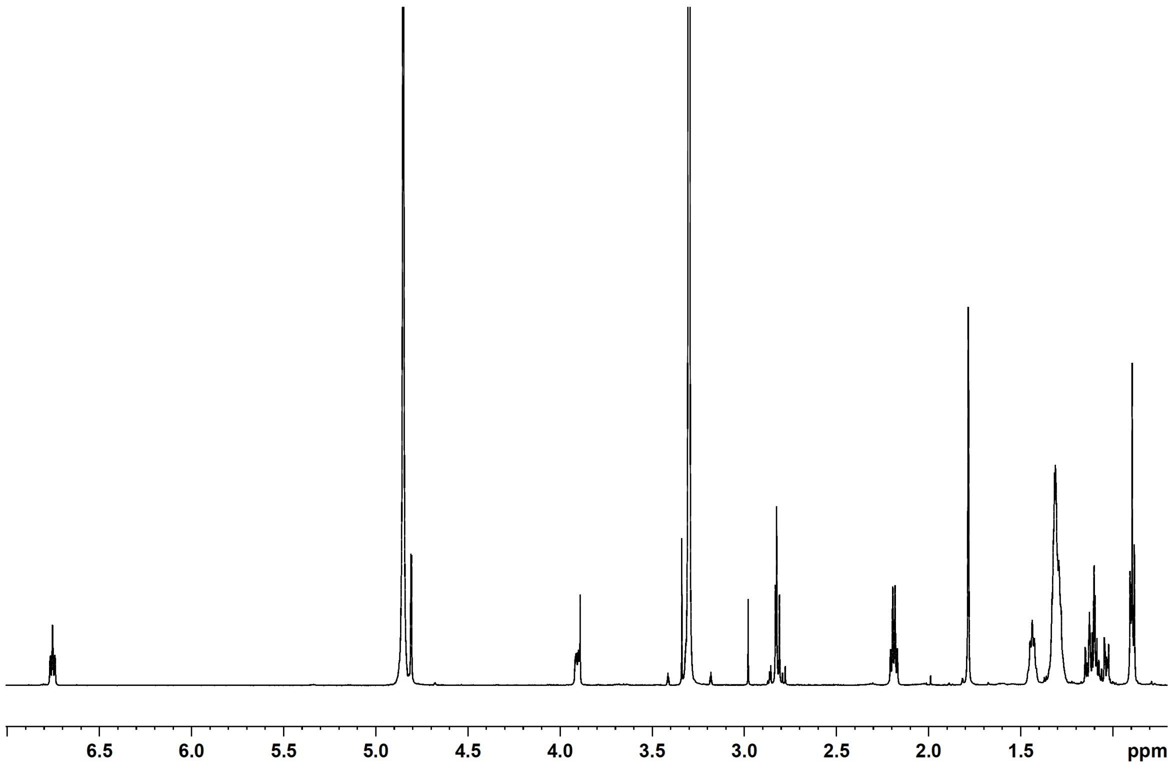

Supplementary Figure 27. ^{1}H NMR spectrum of **3** (CD_3OD).

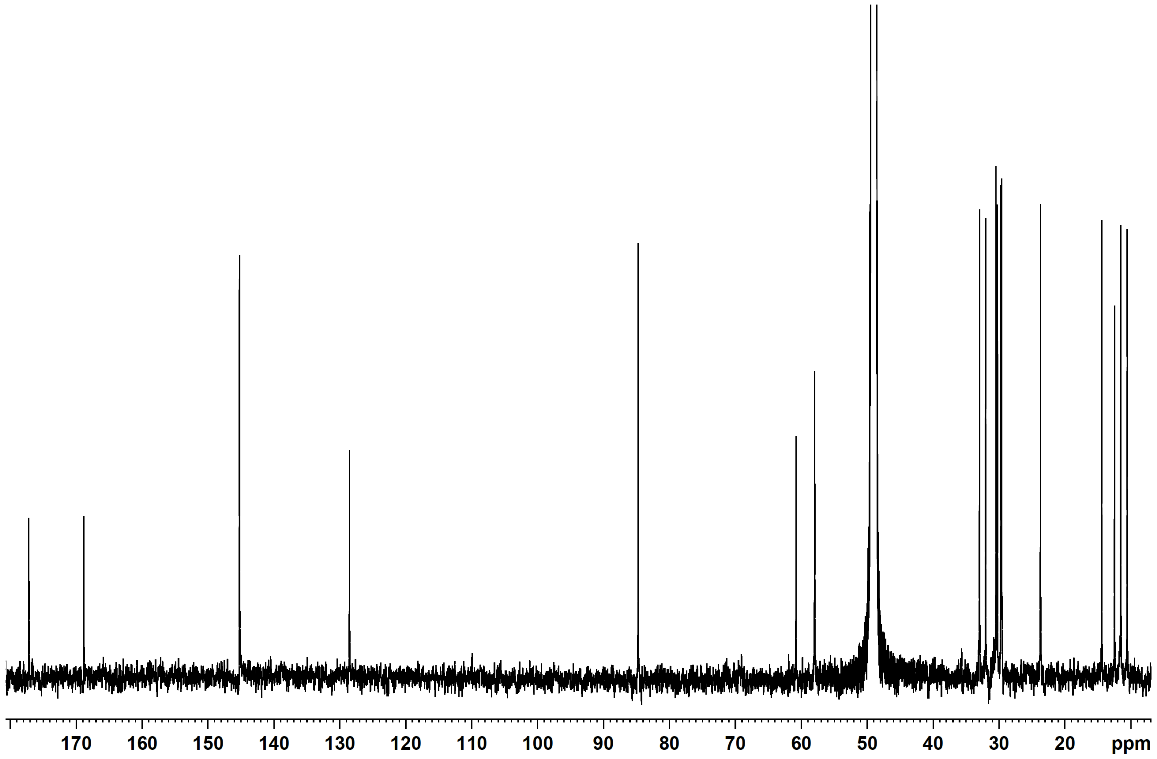

Supplementary Figure 28. ^{13}C NMR spectrum of **3** (CD_3OD).

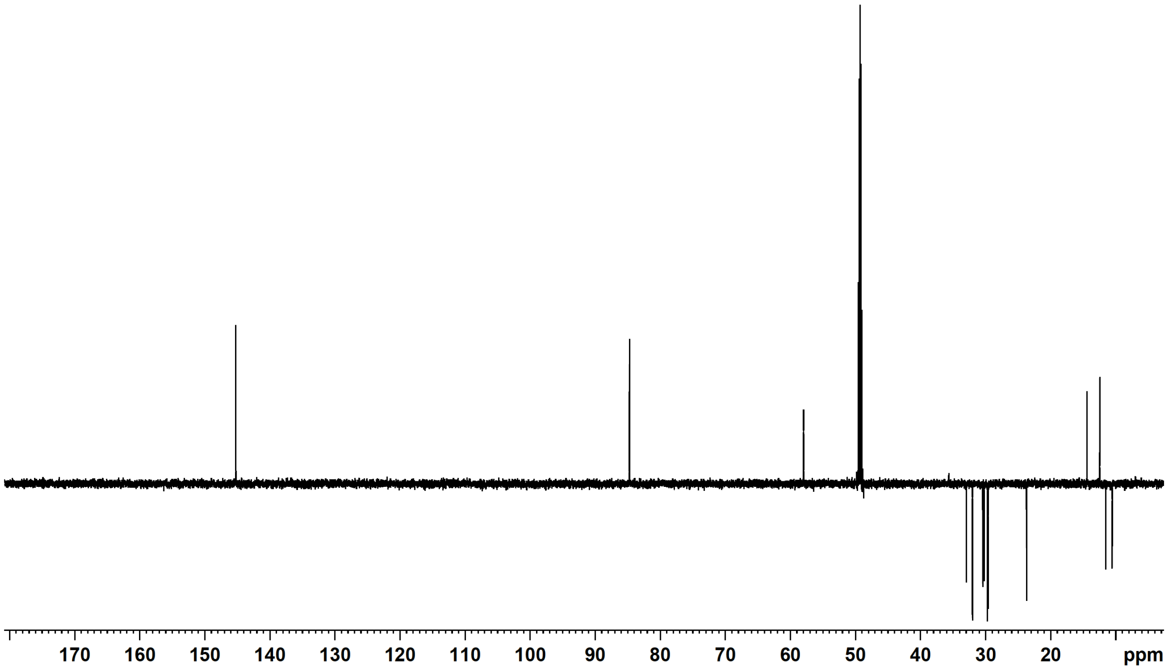

Supplementary Figure 29. DEPT135 spectrum of **3** (CD_3OD).

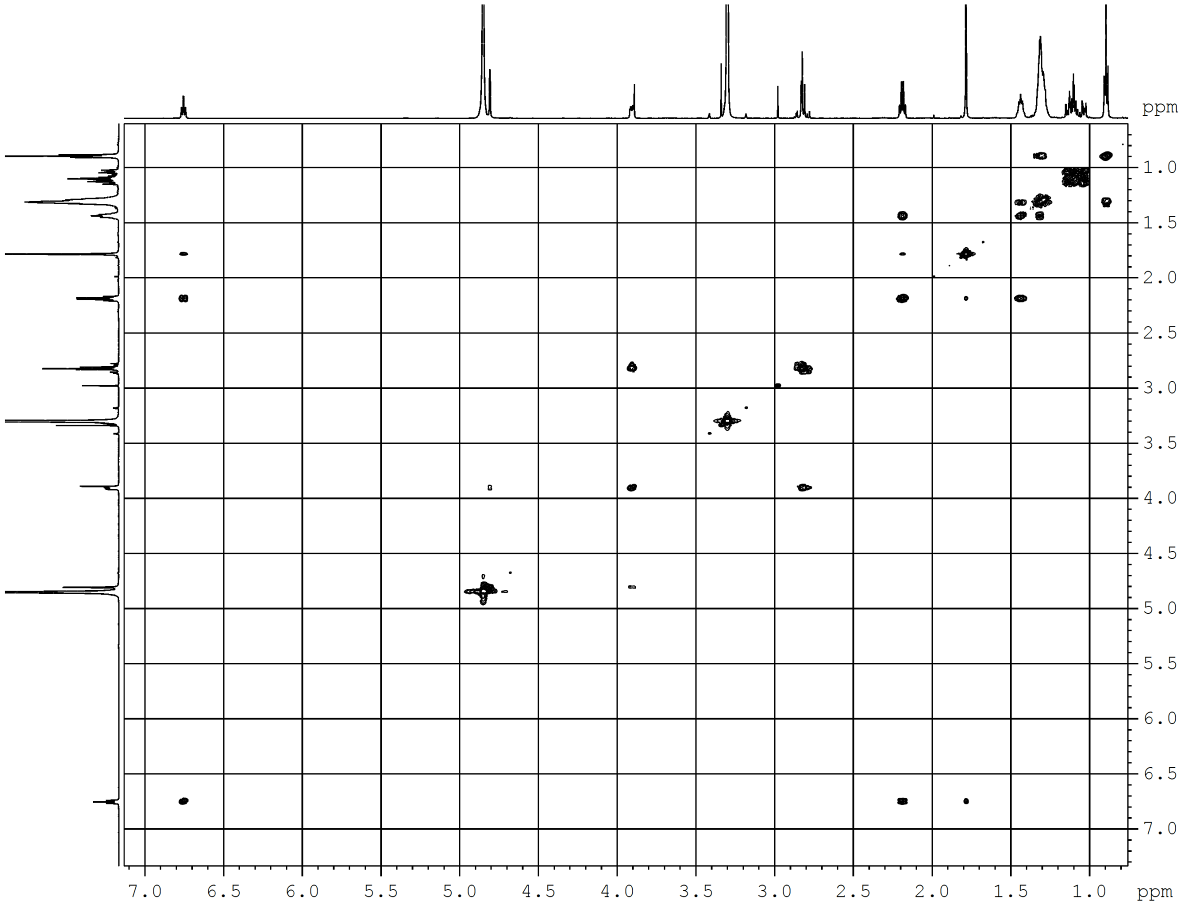

Supplementary Figure 30. COSY spectrum of **3** (CD_3OD).

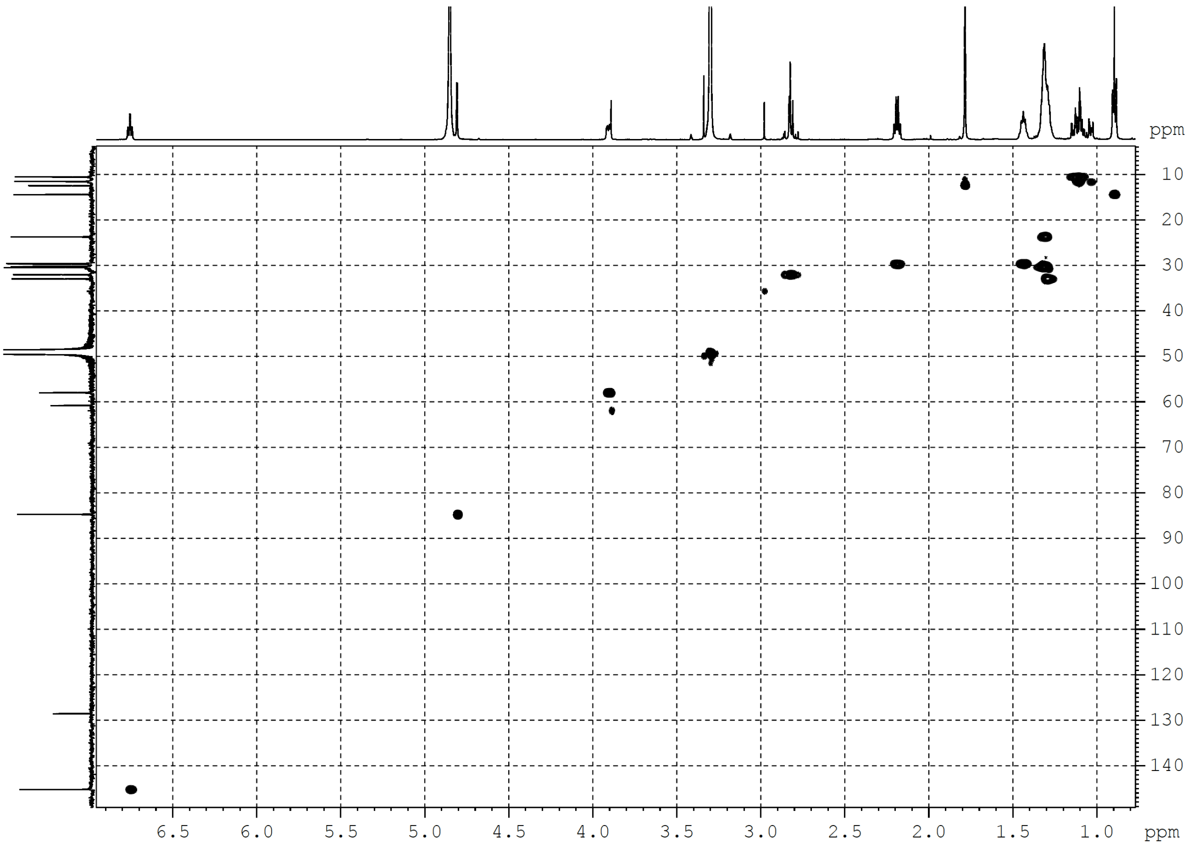

Supplementary Figure 31. HSQC spectrum of **3** (CD_3OD).

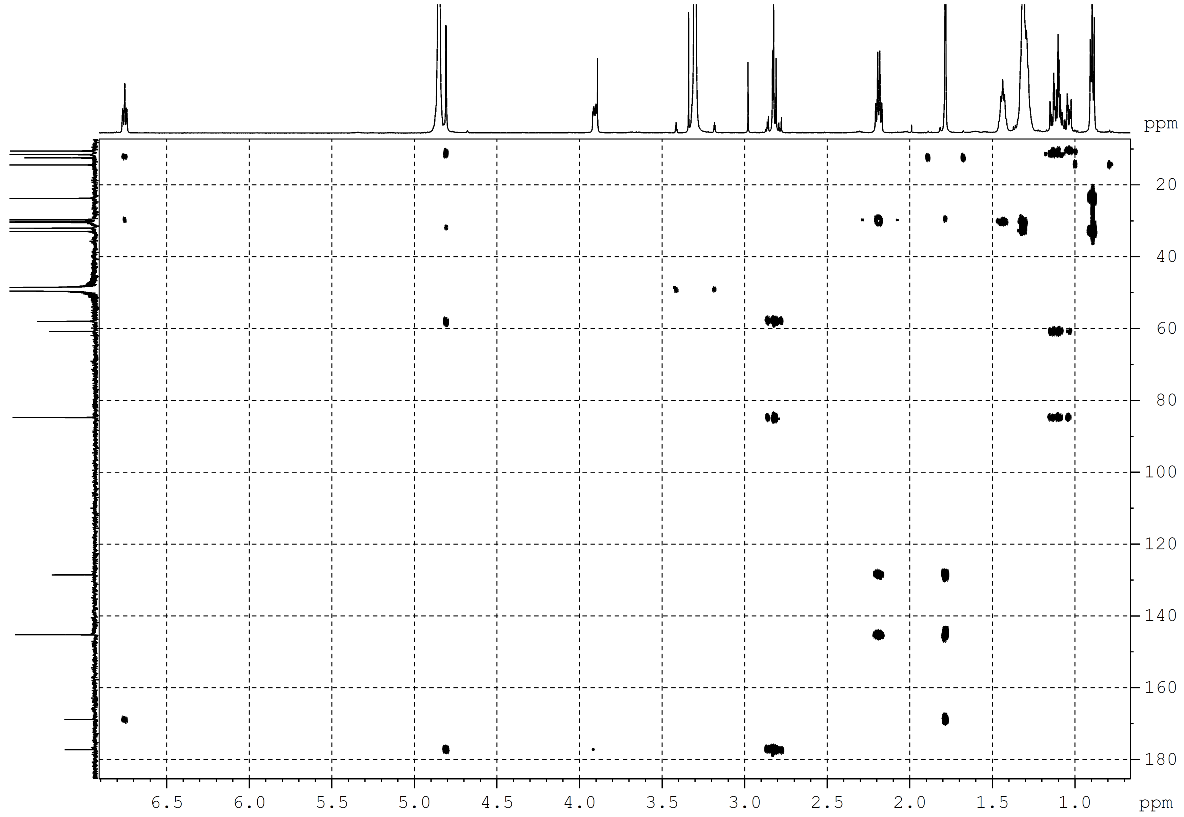

Supplementary Figure 32. HMBC spectrum of **3** (CD_3OD).

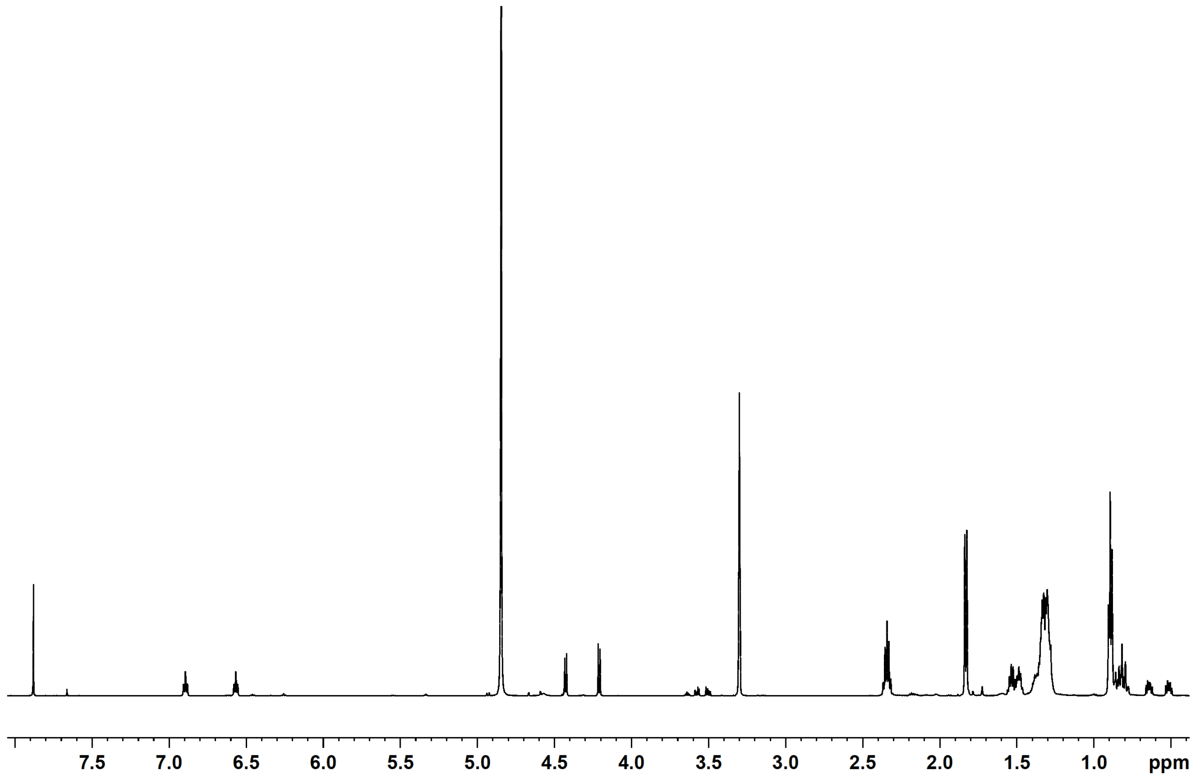

Supplementary Figure 33. ^{1}H NMR spectrum of **4a** (CD_3OD).

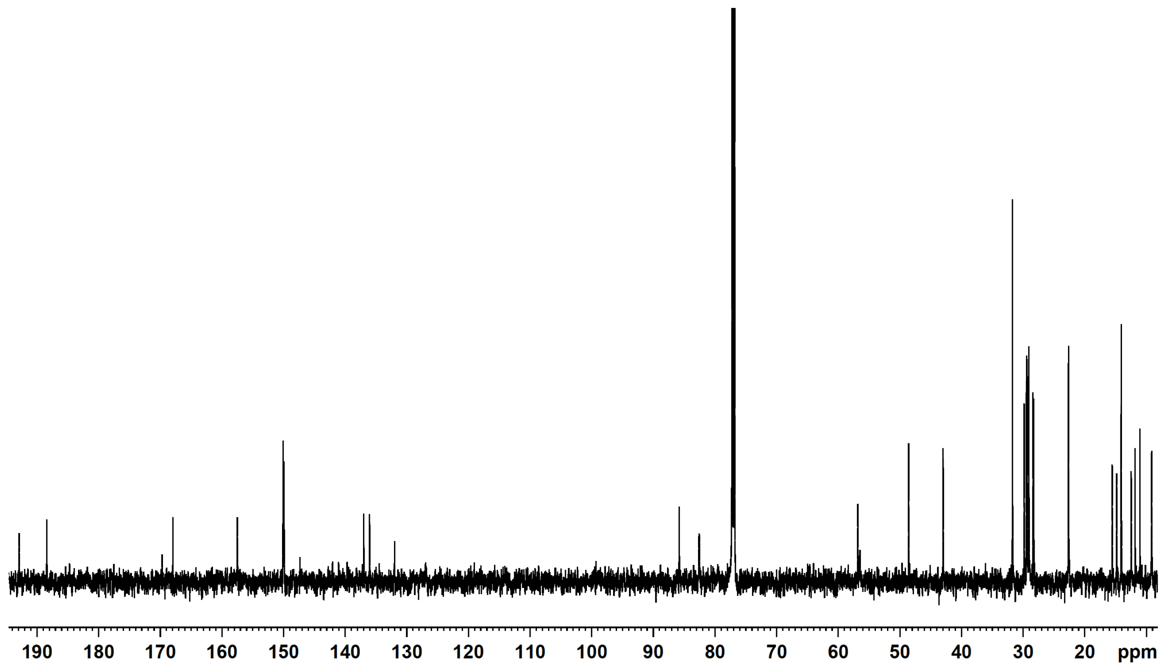

Supplementary Figure 34. ^{13}C NMR spectrum of **4a** (CD_3OD).

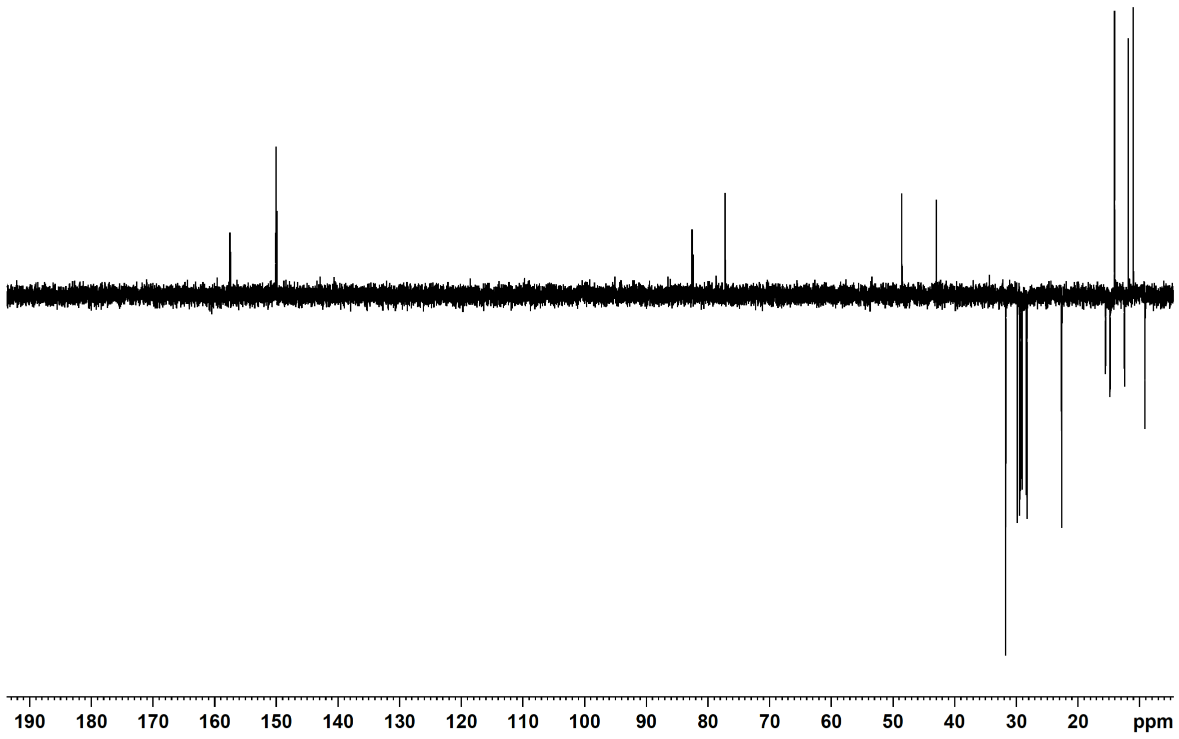

Supplementary Figure 35. DEPT135 spectrum of **4a** (CD_3OD).

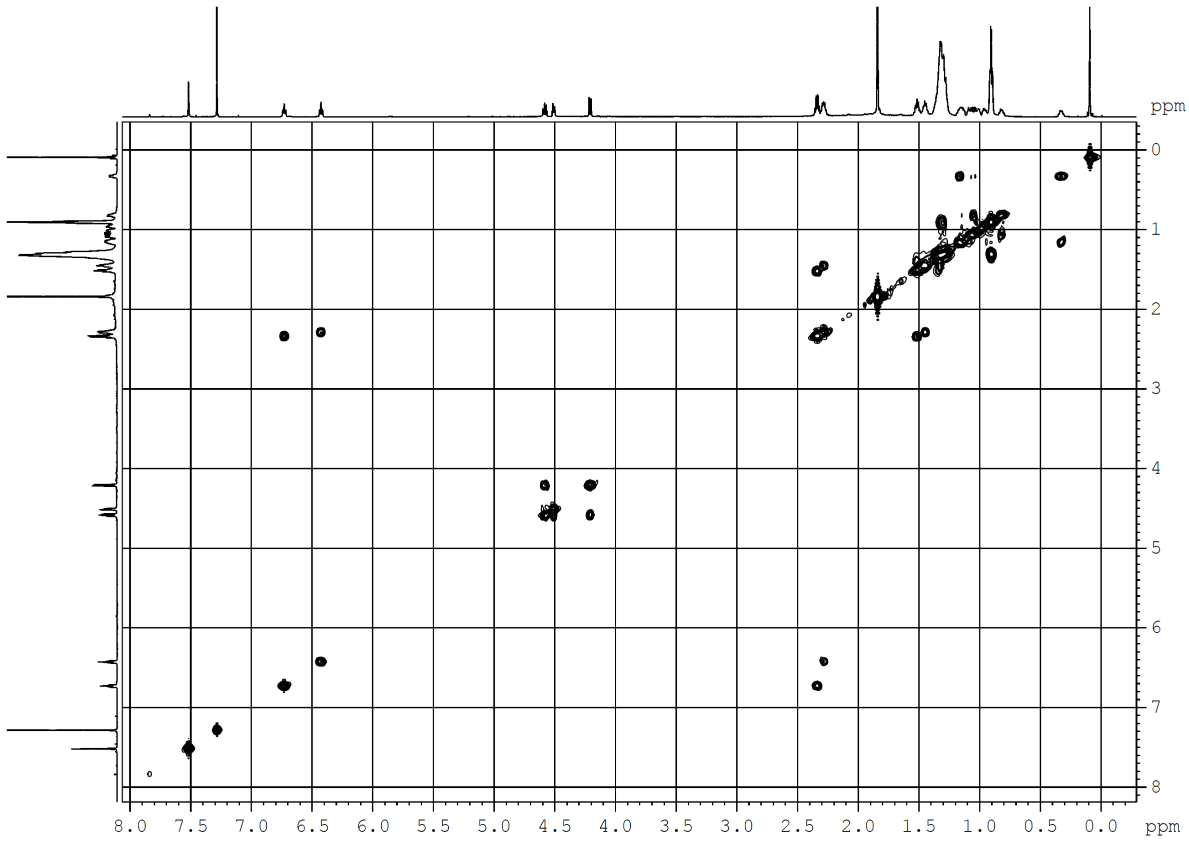

Supplementary Figure 36. COSY spectrum of **4a** (CD_3OD).

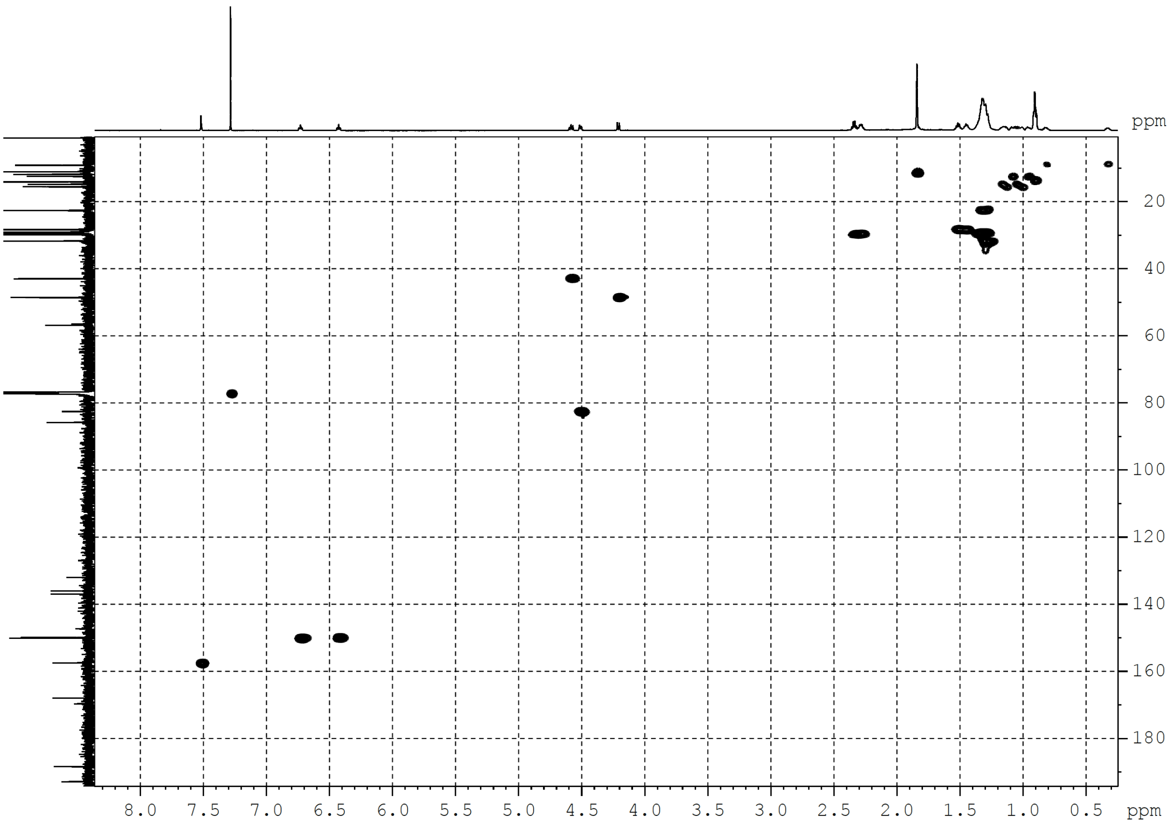

Supplementary Figure 37. HSQC spectrum of **4a** (CD_3OD).

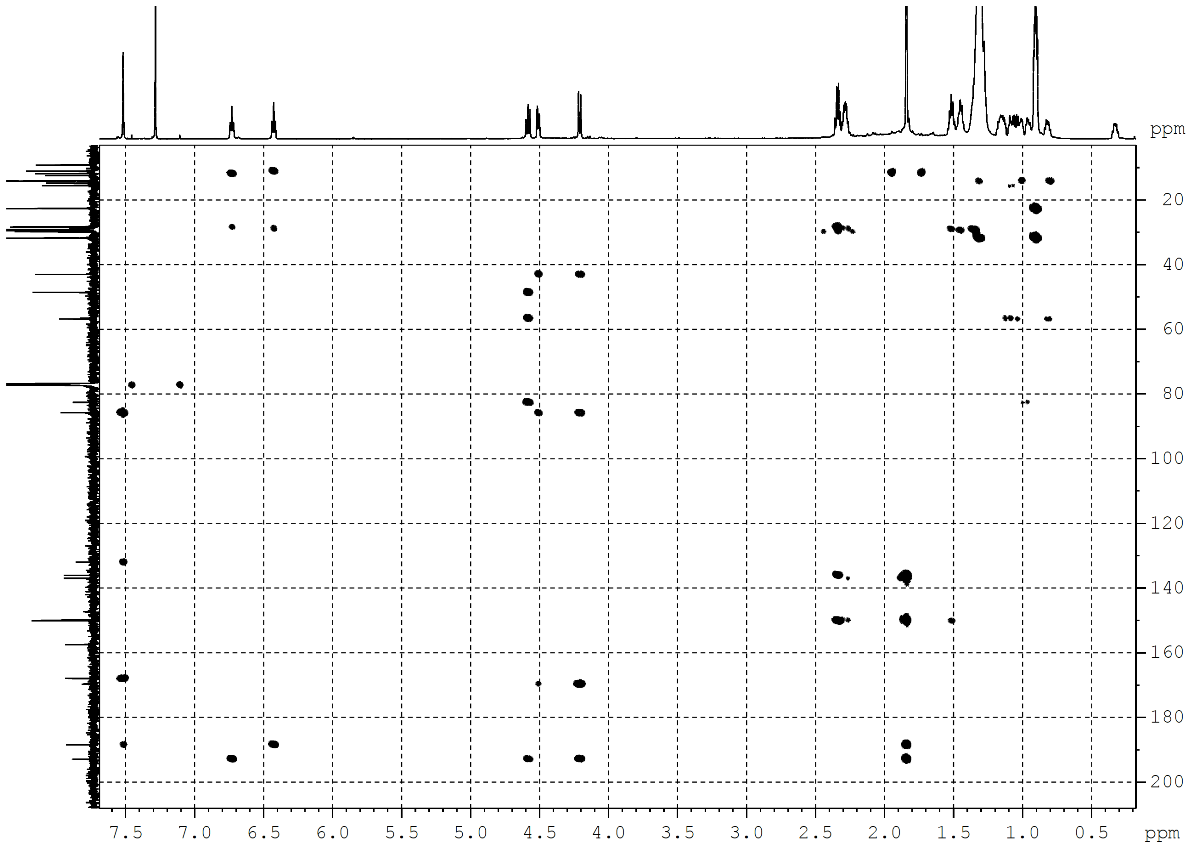

Supplementary Figure 38. HMBC spectrum of **4a** (CD_3OD).

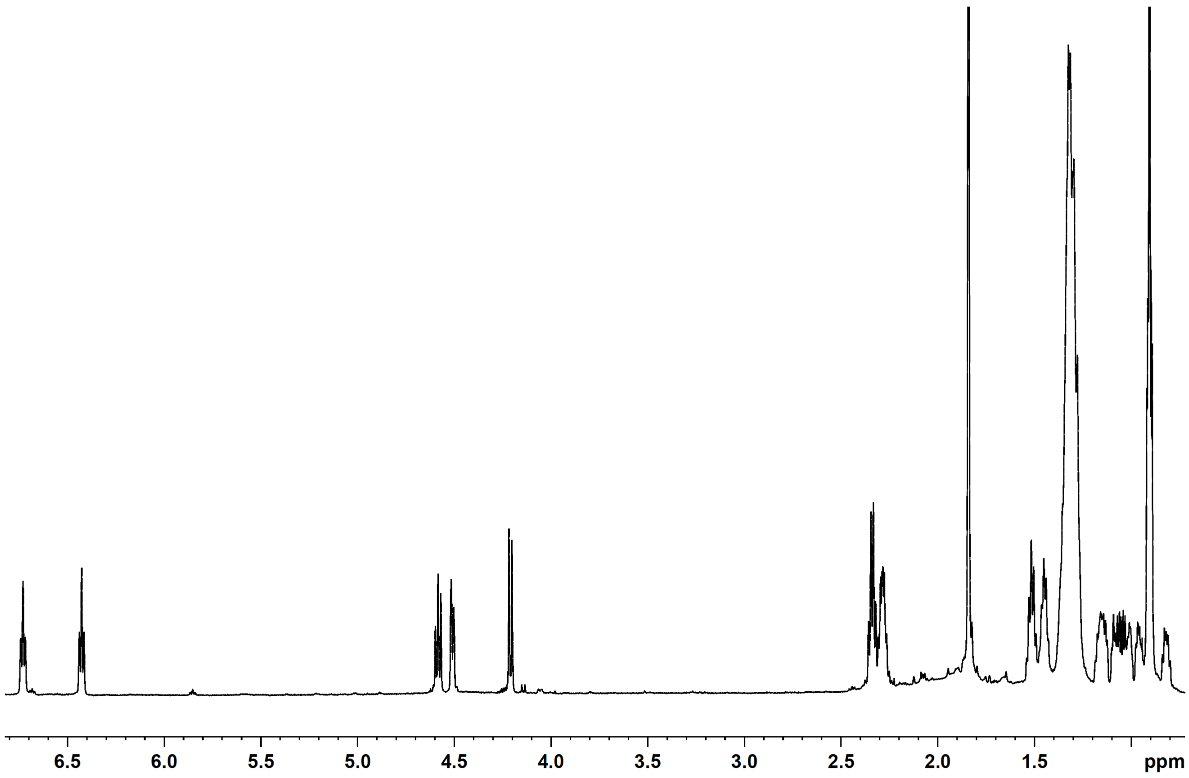

Supplementary Figure 39. ^{1}H NMR spectrum of **4a** ($CDCl_3$).

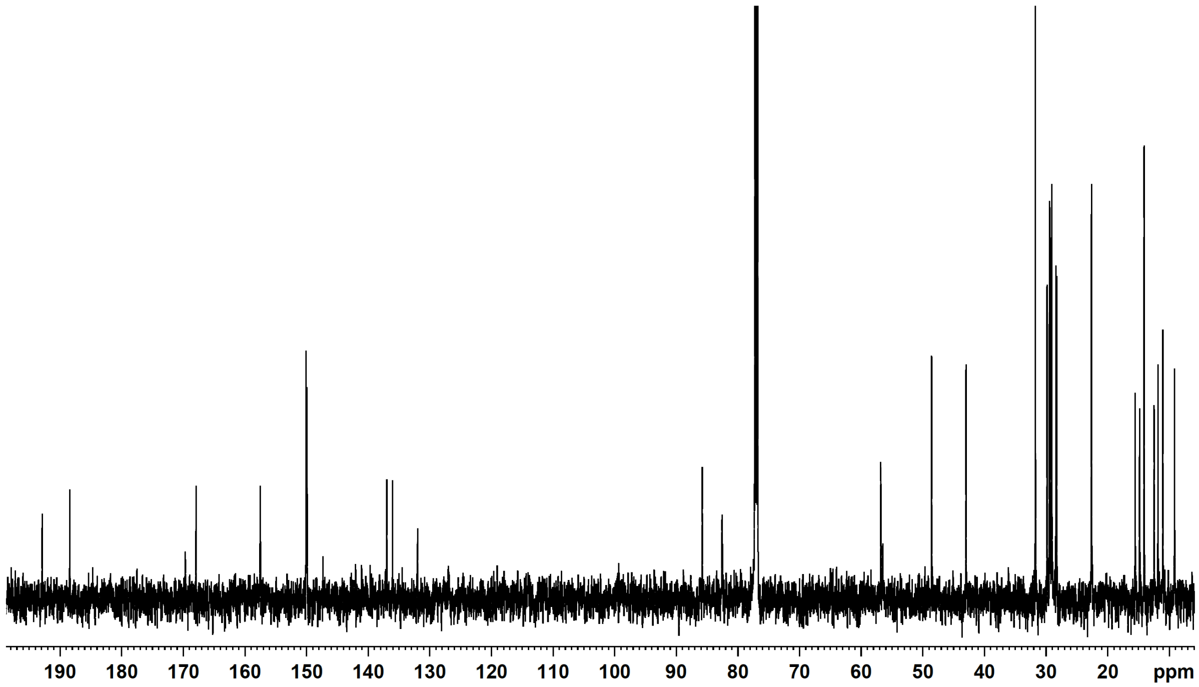

Supplementary Figure 40. ^{13}C NMR spectrum of **4a** ($CDCl_3$).

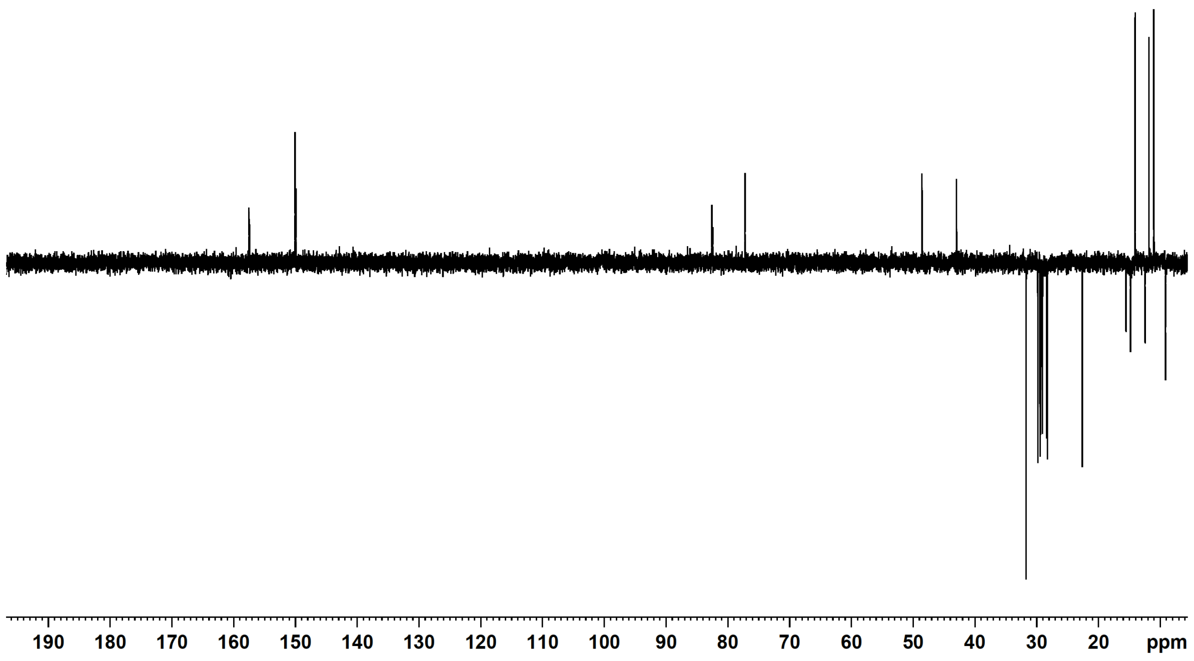

Supplementary Figure 41. DEPT135 spectrum of **4a** ($CDCl_3$).

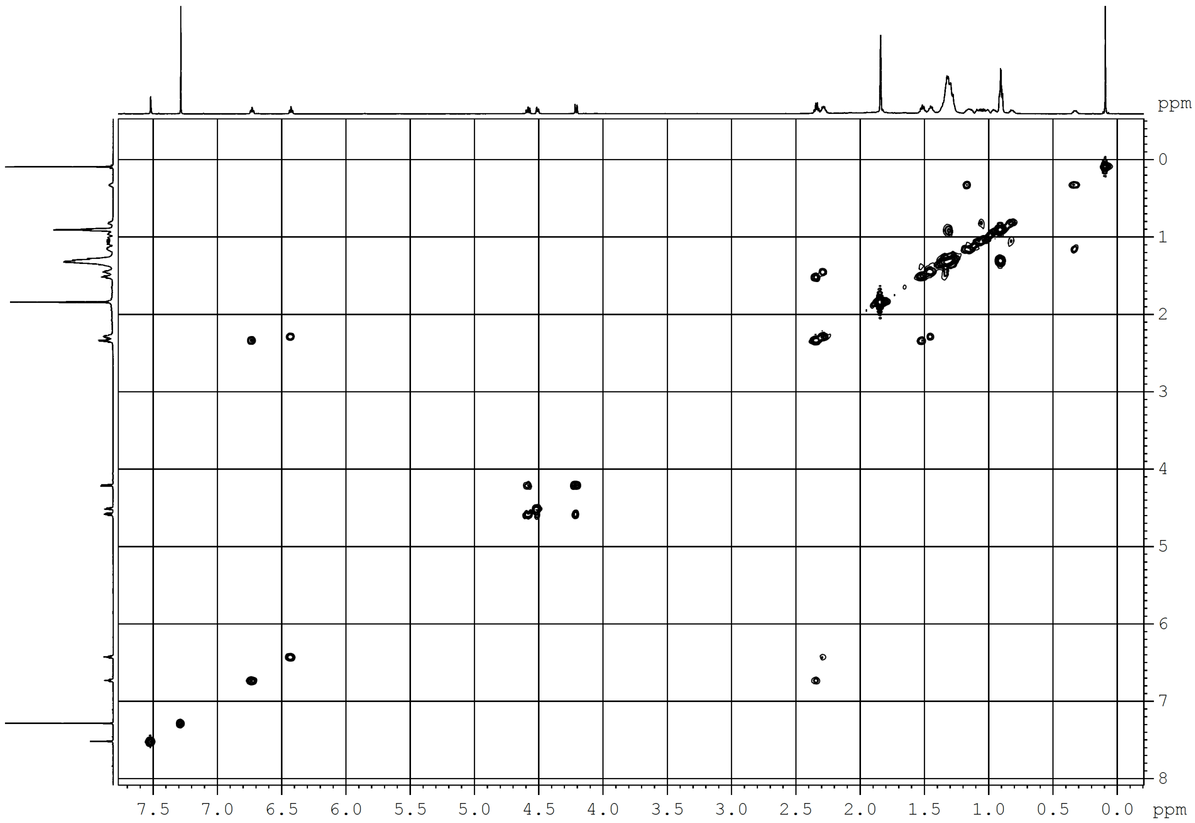

Supplementary Figure 42. COSY spectrum of **4a** ($CDCl_3$).

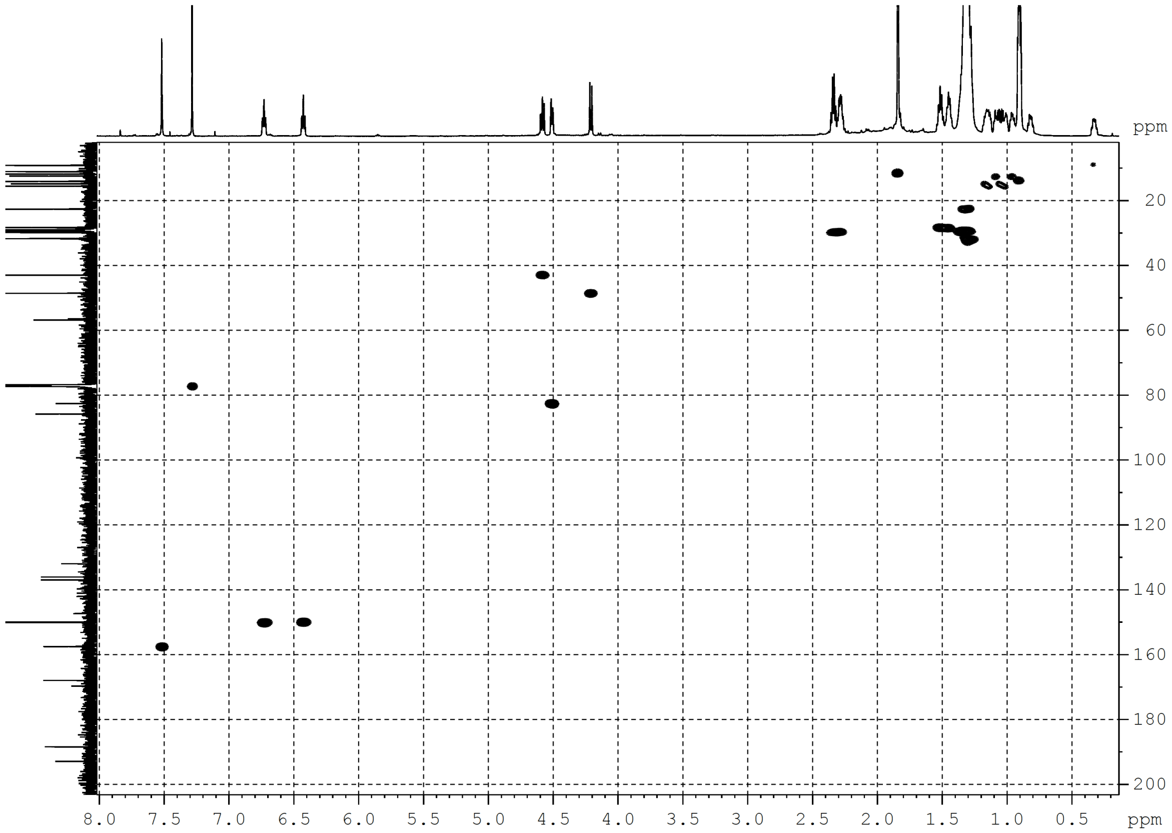

Supplementary Figure 43. HSQC spectrum of **4a** ($CDCl_3$).

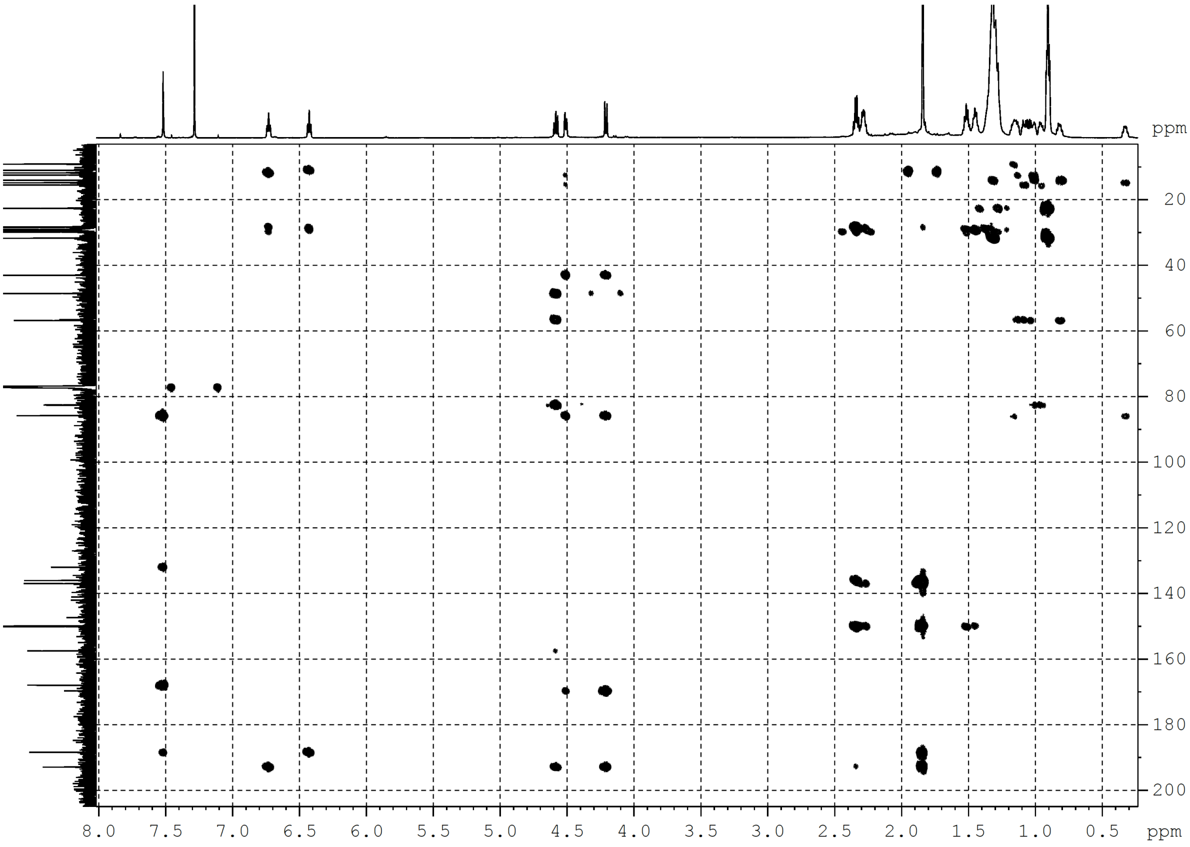

Supplementary Figure 44. HMBC spectrum of **4a** ($CDCl_3$).

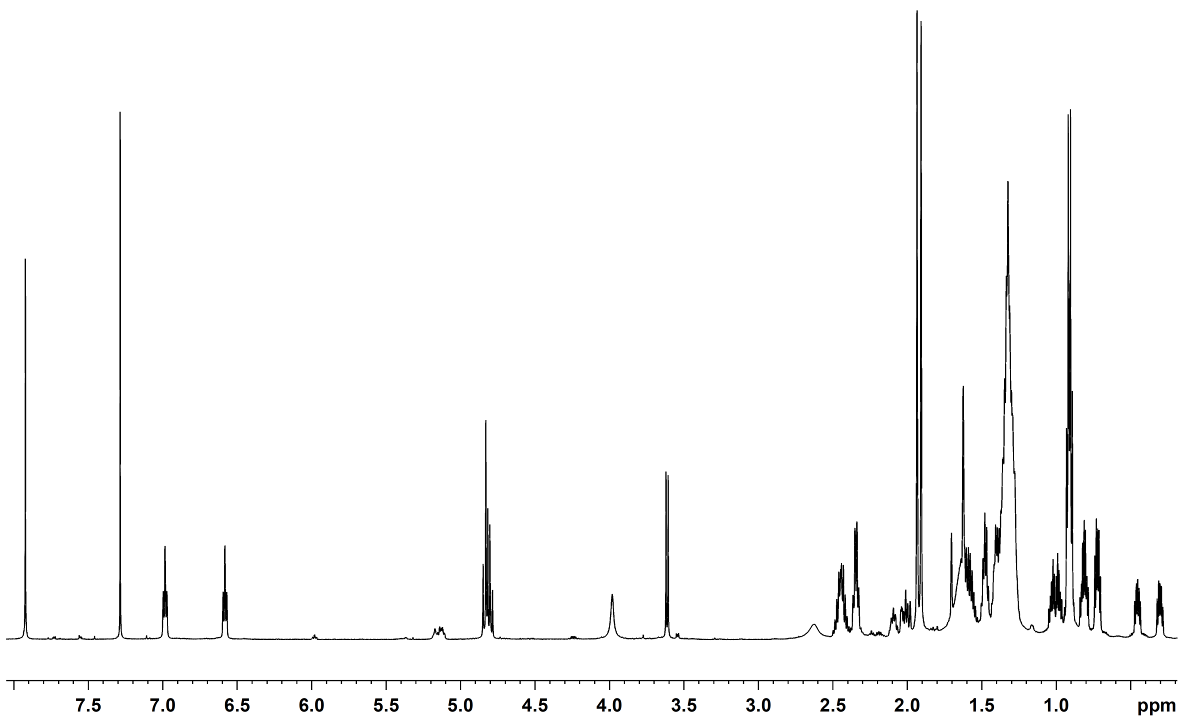

Supplementary Figure 45. ^{1}H NMR spectrum of **4b** ($CDCl_3$).

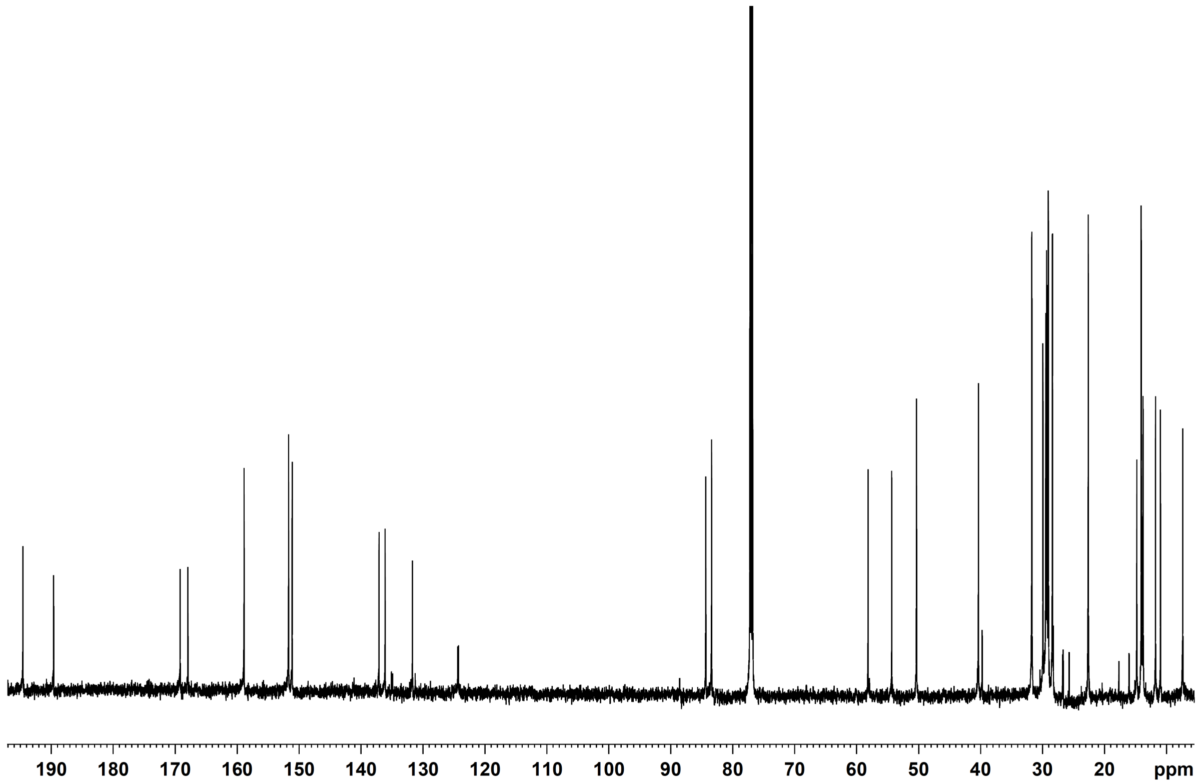

Supplementary Figure 46. ^{13}C NMR spectrum of **4b** ($CDCl_3$).

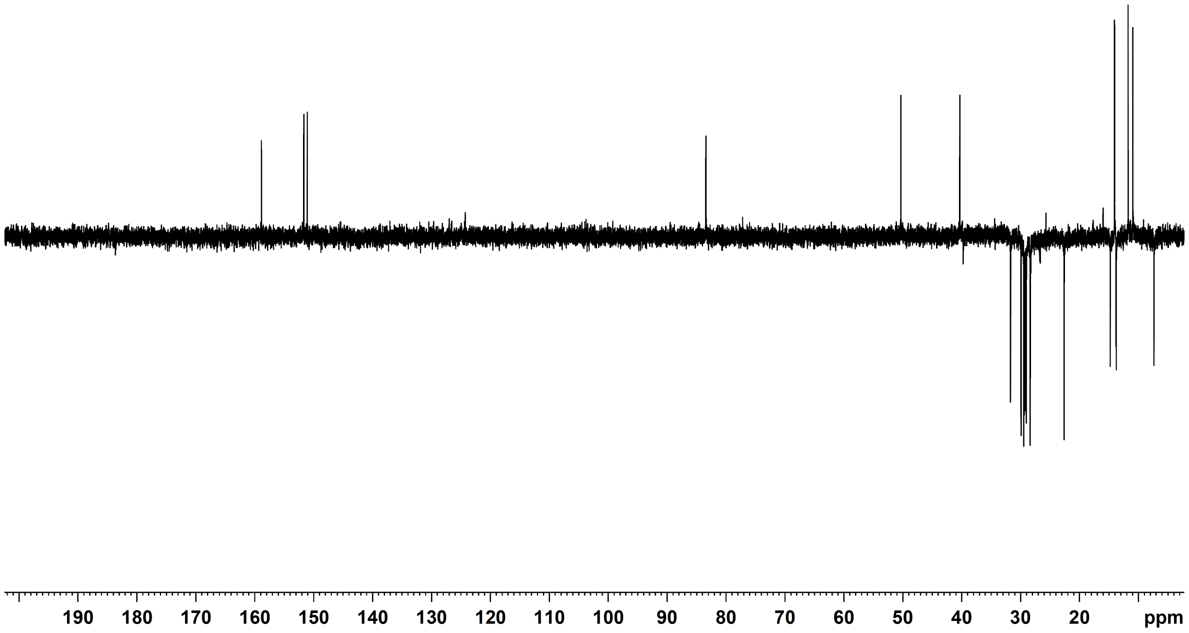

Supplementary Figure 47. DEPT135 spectrum of **4b** ($CDCl_3$).

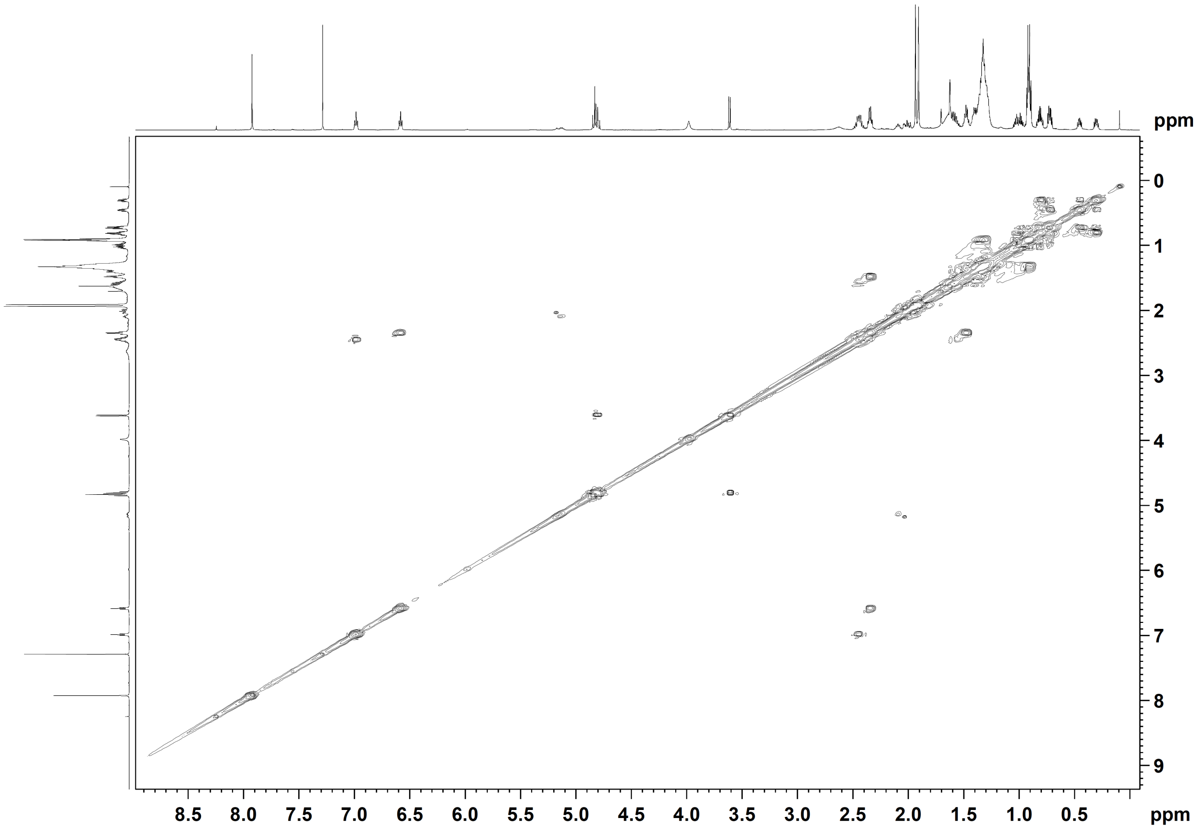

Supplementary Figure 48. COSY spectrum of **4b** ($CDCl_3$).

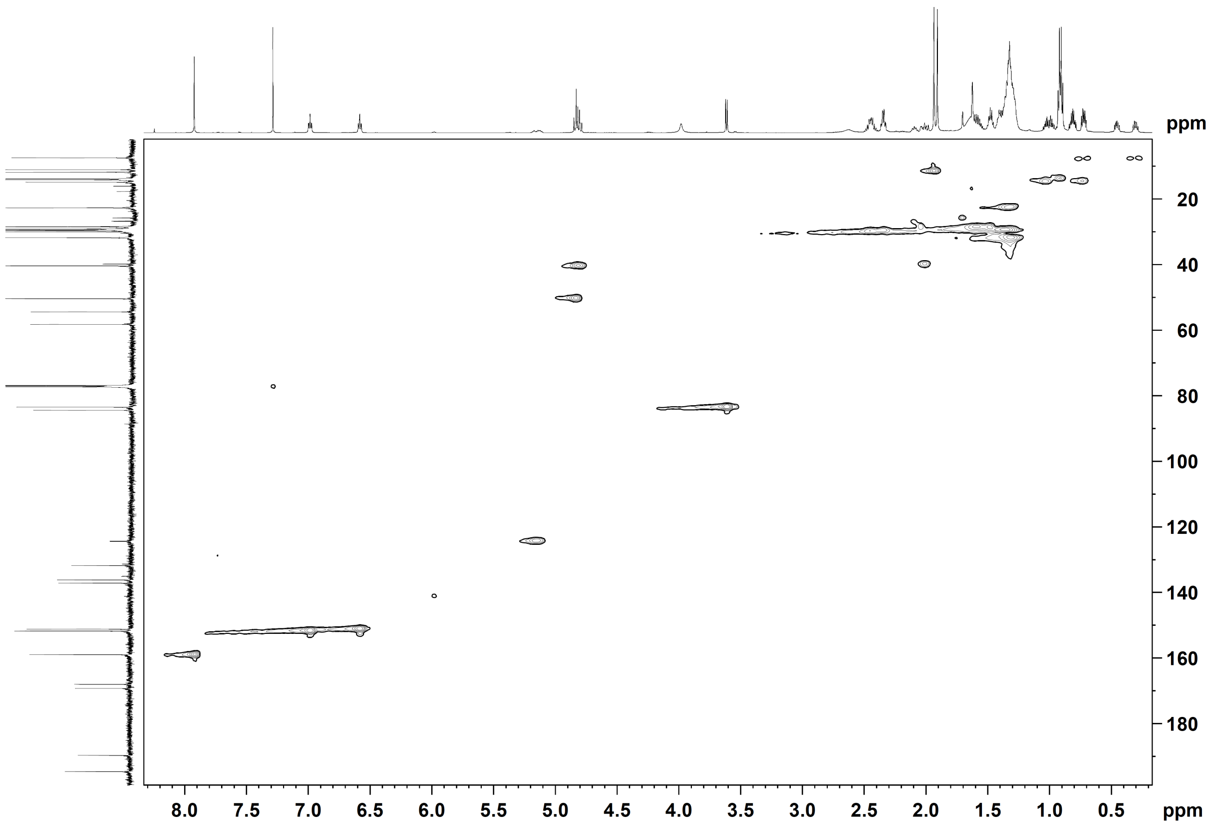

***Supplementary Figure 49*.** HSQC spectrum of **4b** ($CDCl_3$).

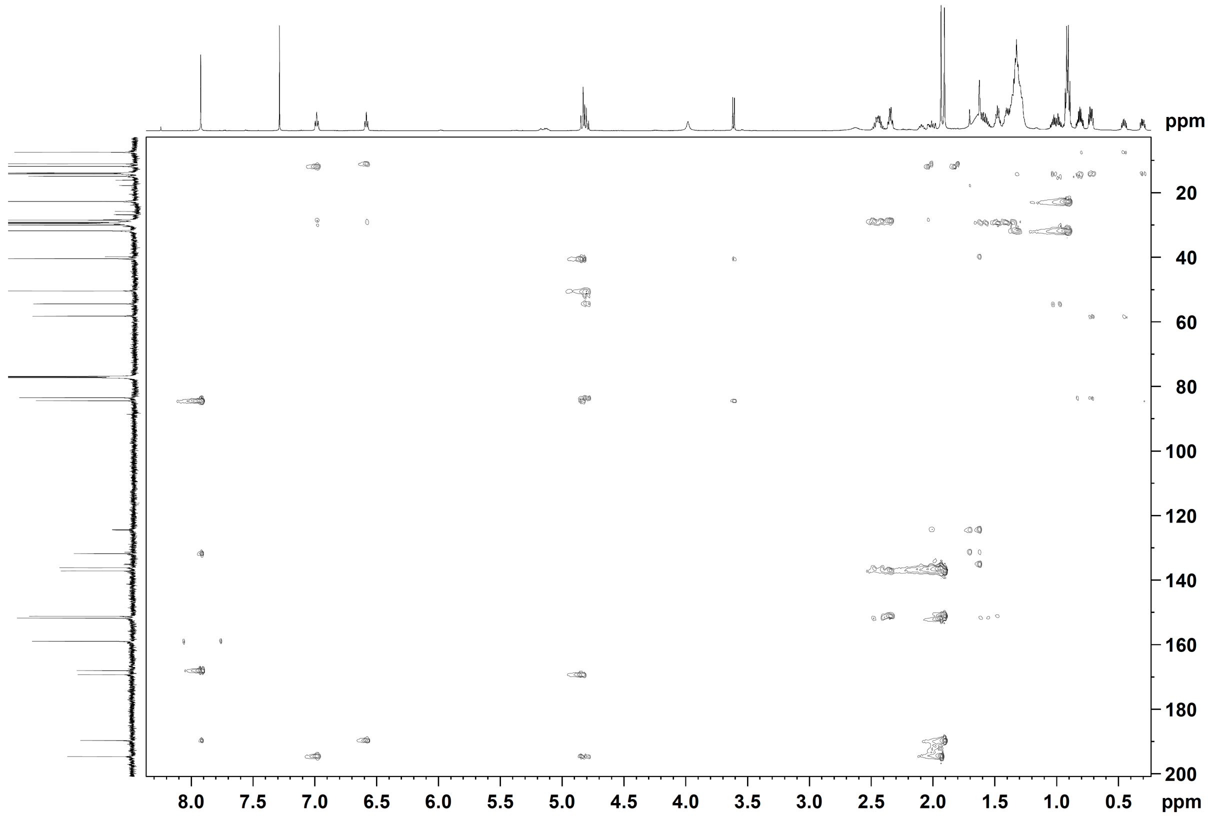

***Supplementary Figure 50*.** HMBC spectrum of **4b** ($CDCl_3$).

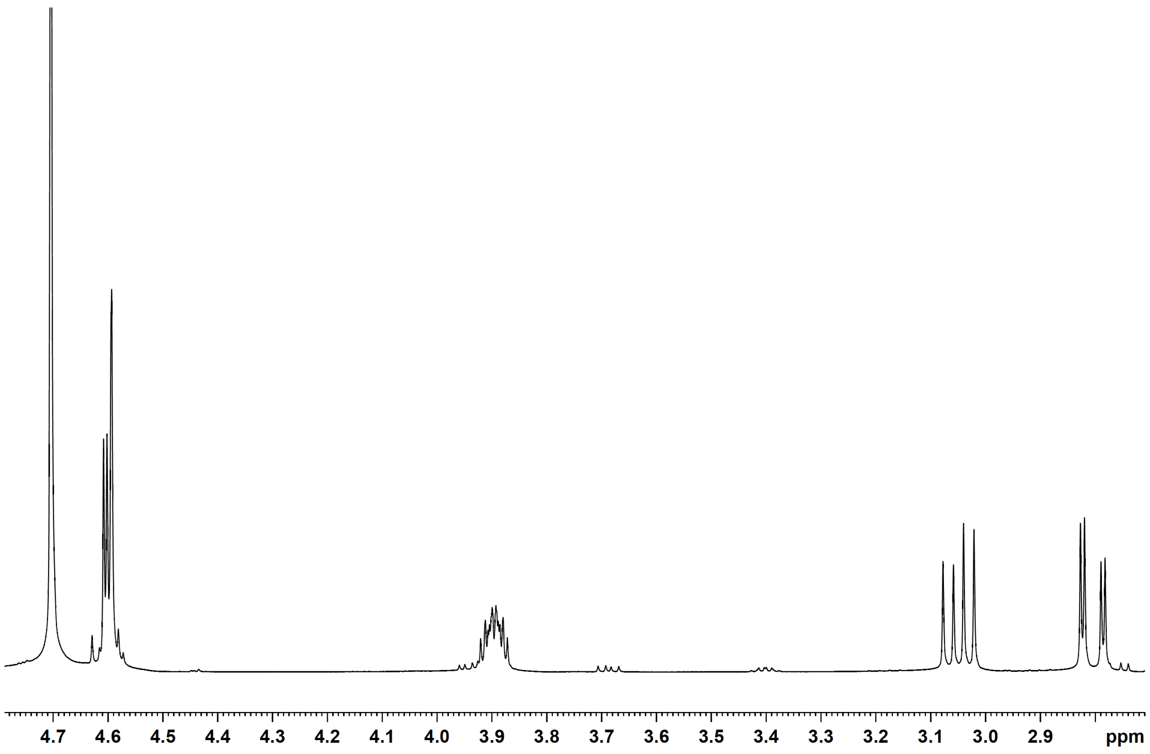

Supplementary Figure 51. ^{1}H NMR spectrum of γ-butyrolactone-3-sulfonic acid (D_2O).

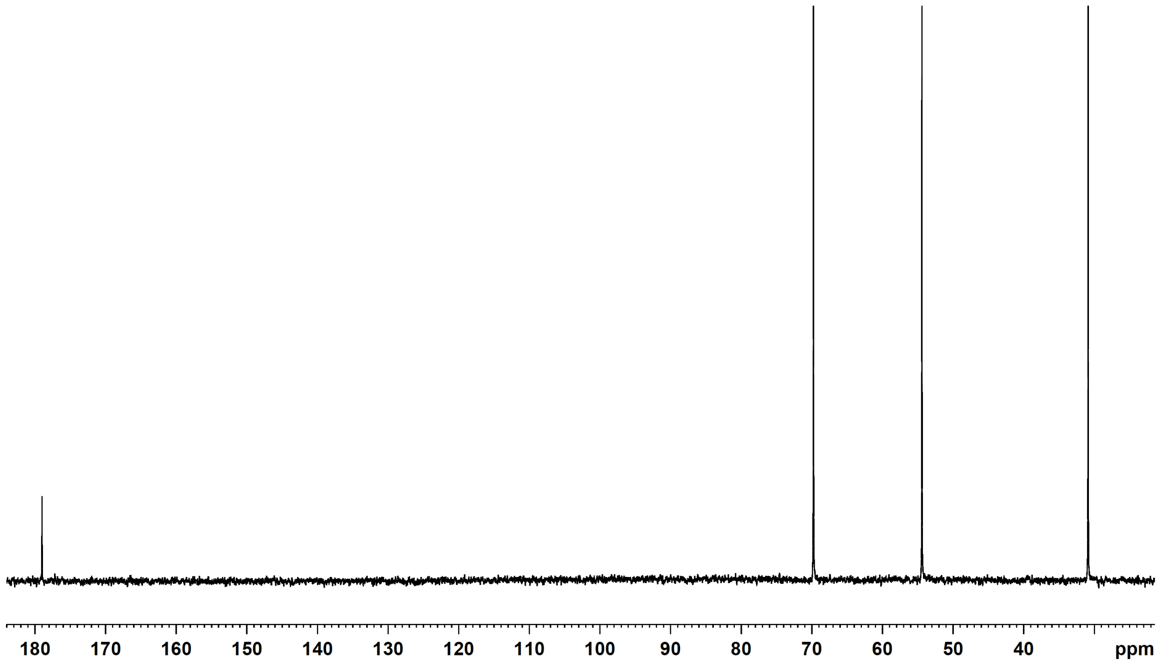

Supplementary Figure 52. ^{13}C NMR spectrum of γ-butyrolactone-3-sulfonic acid (D_2O).

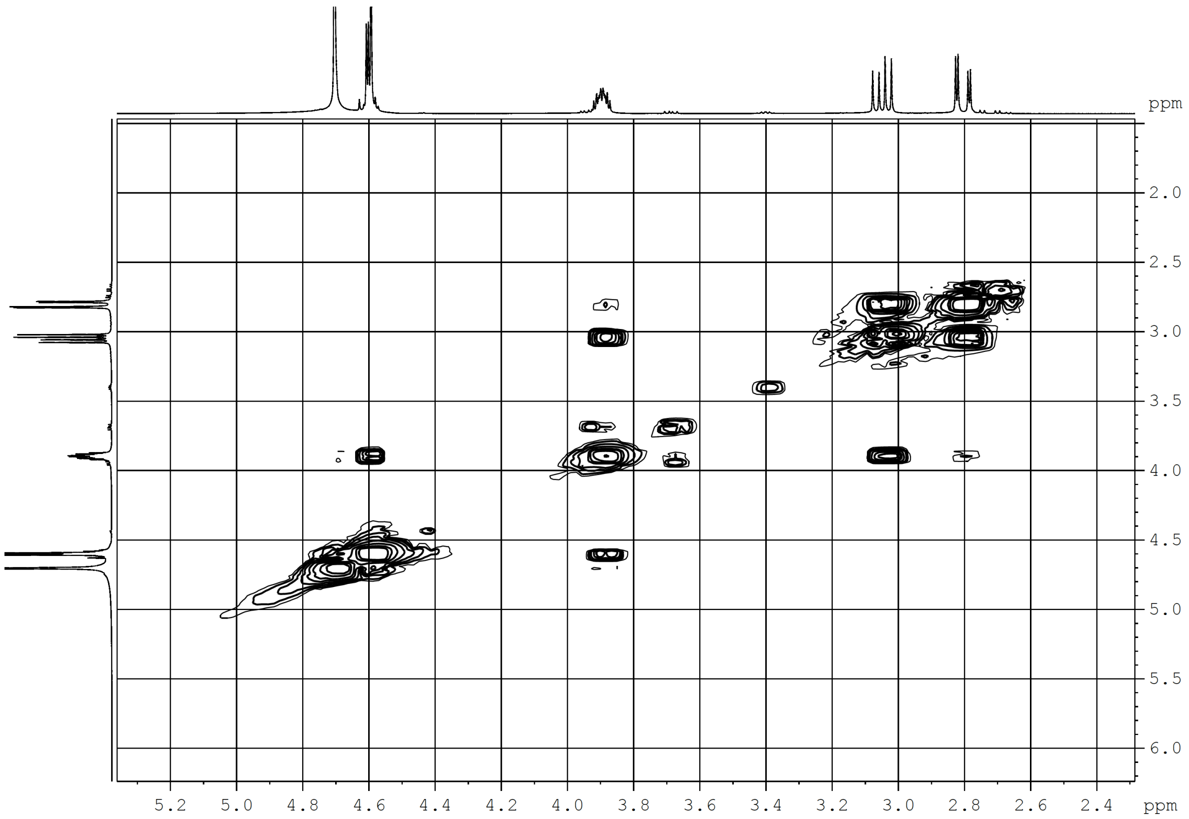

Supplementary Figure 53. COSY spectrum of γ-butyrolactone-3-sulfonic acid (D_2O).

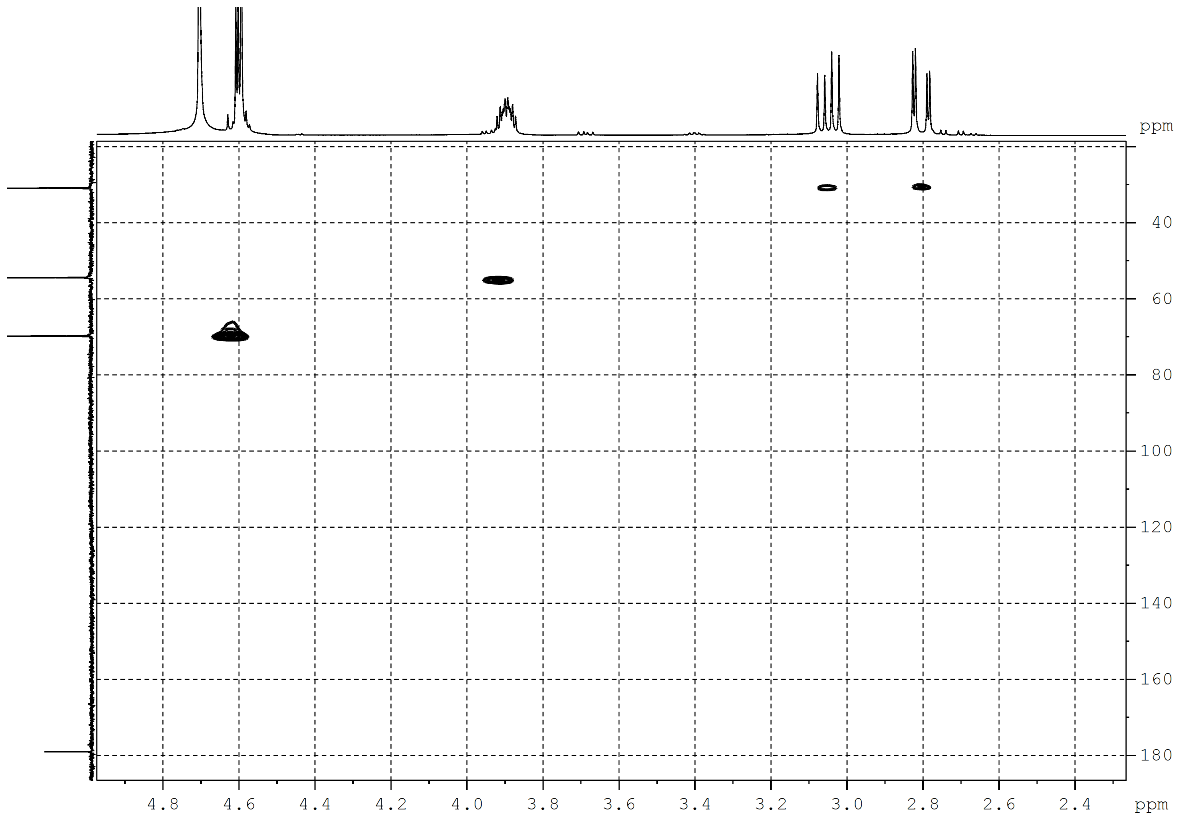

Supplementary Figure 54. HSQC spectrum of γ-butyrolactone-3-sulfonic acid (D_2O).

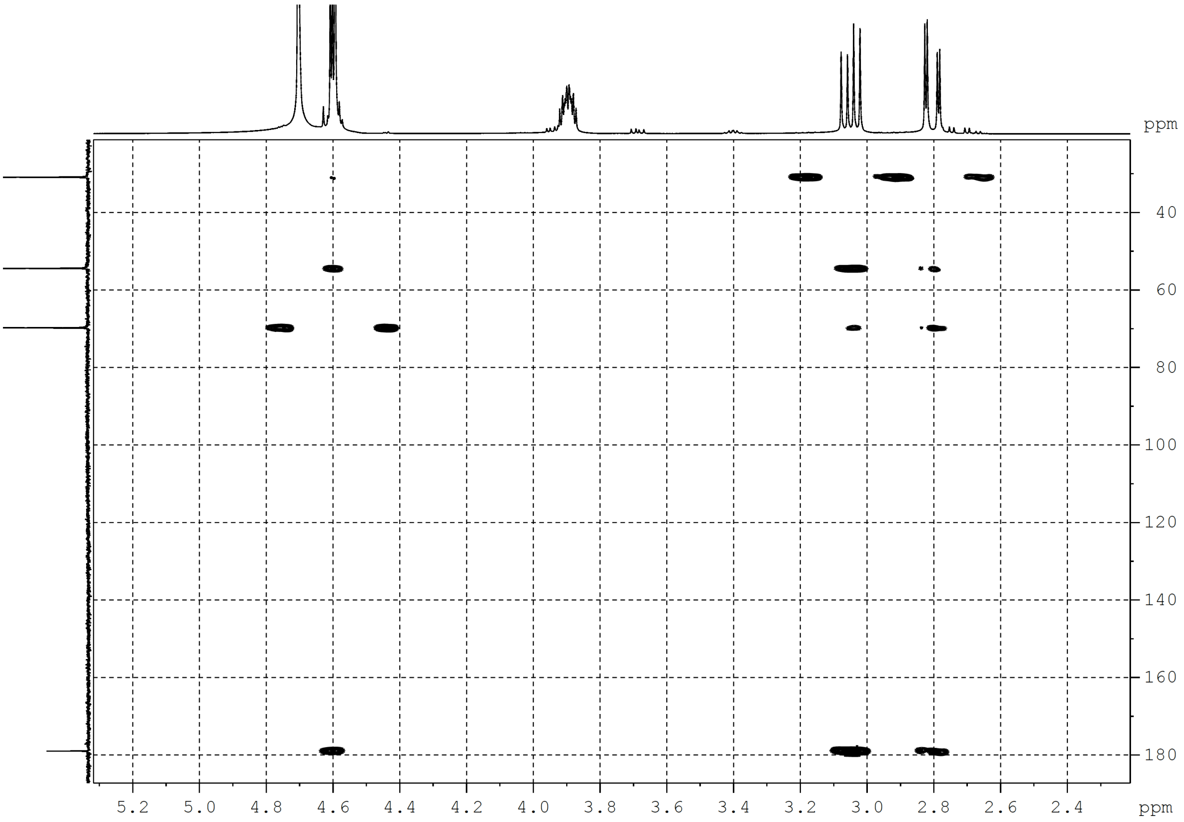

Supplementary Figure 55. HMBC spectrum of γ-butyrolactone-3-sulfonic acid (D_2O).

Supplemental References

[1] M. Roudier, T. Constantieux, A. Quintard, J. Rodriguez, *Org. Lett.* **2014**, *16*, 2802-2805.

[2] A. El Bouakher, R. Le Goff, J. Tasserie, J. Lhoste, A. Martel, S. Comesse, *Org. Lett.* **2016**, *18*, 2383-2386.

[3] X. He, J. Li, H. Gao, F. Qiu, X. Cui, X. Yao, *Chem. Pharm. Bull.* **2003**, *51*, 586-589.

[4] J. Sulston, J. Hodgkin, *The Nematode Caenorhabdtitis elegans, Vol. 17*, Cold Spring Harbor Laboratory Press, Cold Spring Harbor, NY, **1988**.

[5] L. Byerly, R. C. Cassada, R. L. Russell, *Rev. Sci. Instrum.* **1975**, *46*, 517-522.

[6] K. Meier, W. Klöckner, B. Bonhage, E. Antonov, L. Regestein, J. Büchs, *Biochem. Eng. J.* **2016**, *109*, 228-235.

[7] R. D. Pridmore, *Gene* **1987**, *56*, 309-312.

[8] G. Lackner, N. Moebius, C. Hertweck, *The ISME Journal* **2011**, *5*, 252-261.

[9] J. Franke, K. Ishida, C. Hertweck, *Angew. Chem. Int. Ed.* **2012**, *51*, 11611-11615.

[10] S. Yllner, *Acta Chem. Scand.* **1956**, *10*, 1251-1256.

[11] K. Scherlach, L. P. Partida-Martinez, H.-M. Dahse, C. Hertweck, *J. Am. Chem. Soc.* **2006**, *128*, 11529-11536.

[12] X. Huajiang, P. Catherine, W. Alison, *Sci. Rep.* **2017**, *7*, 983-989.
